# Determination and Analysis of Thermodynamic Properties of Methyl Methylanthranilate Isomers

**DOI:** 10.3390/molecules28186686

**Published:** 2023-09-18

**Authors:** Carlos A. O. Silva, Vera L. S. Freitas, Maria D. M. C. Ribeiro da Silva

**Affiliations:** Research Center, Institute of Molecular Sciences (IMS), Department of Chemistry and Biochemistry (DQB), Faculty of Sciences, University of Porto (FCUP), Rua do Campo Alegre, 4169-007 Porto, Portugalmdsilva@fc.up.pt (M.D.M.C.R.d.S.)

**Keywords:** fragrance compounds, energetic study, enthalpy of combustion, enthalpy of phase transition, enthalpy of formation, methyl substituent, contaminants

## Abstract

The enthalpies of formation in the gaseous phase of methyl 3-methylanthranilate and methyl 5-methylanthranilate were determined from experimental measurements of the corresponding standard energies of combustion, obtained from combustion calorimetry, and the standard enthalpies of vaporization and sublimation, obtained from Calvet microcalorimetry and Knudsen mass-loss effusion. A computational study, using the G3(MP2)//B3LYP composite method, has also been performed for the calculation of the gas-phase standard enthalpies of formation of those two molecules at *T* = 298.15 K, as well as for the remaining isomers, methyl 4-methylanthranilate and methyl 6-methylanthranilate. The results have been used to evaluate and analyze the energetic effect of the methyl substituent in different positions of the ring.

## 1. Introduction

Fragrances are ingredients present in many consumer products, primarily used to enhance their scent and influence consumer purchasing decisions. However, these substances can have negative environmental impacts as they persist and accumulate in various ecosystems due to the inefficiency of conventional wastewater treatment processes in removing them from treated water before their discharge into effluents. Environmentalists have raised concerns about the potential long-term effects of fragrance accumulation on ecosystems [1,2,3,4,5,6]. In cases where conventional treatments are unable to eliminate micropollutants, closer environmental risk monitoring of fragrances prior to their commercial use can be an effective solution. Accurate thermodynamic data are essential to correctly predicting the distribution of fragrances in environmental compartments. However, it has been observed that regulatory authorities often rely on estimated values obtained from redundant forecasting schemes, which greatly reduces their reliability.

**Figure 1:**
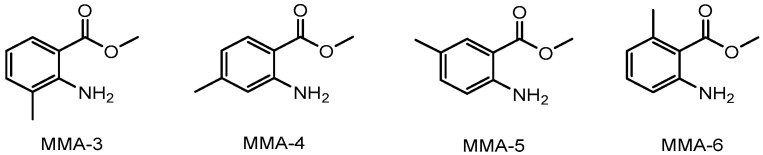
The molecular formulae of methyl 3-methylanthranilate (MMA-3), methyl 4-methylanthranilate (MMA-4), methyl 5-methylanthranilate (MMA-5), and methyl 6-methylanthranilate (MMA-6).

This work is part of a broader research project that aims to determine the thermodynamic properties of key fragrance classes. The inherent goal is to reduce existing gaps and contribute to the improvement of estimation methods for environmental risk assessment [7,8,9]. A comprehensive comparative analysis of the thermodynamic properties of methyl methylanthranilate (MMA) isomers is presented. The investigated isomers include methyl 3-methyanthranilate (MMA-3), methyl 4-methyanthranilate (MMA-4), methyl 5-methyanthranilate (MMA-5), and methyl 6-methyanthranilate (MMA-6), with molecular formulae depicted in Figure 1.

This research incorporates both computational and practical components. Computational methods were employed to explore the thermodynamic properties of all four isomers, while the practical component focused on MMA-3 and MMA-5. By combining the theoretical calculations and the experimental results, a comprehensive understanding of the thermodynamic characteristics of these methyl methylanthranilate isomers was achieved.

Regarding the MMA-3 and MMA-5 compounds studied experimentally, they are commonly used as food flavorings and fragrance additives due to their characteristic odor [10]. Moreover, MMA-3 is used in orchards and vineyards as a bird deterrent to safeguard crops, while MMA-5 is a chemical intermediate for synthesizing other organic compounds, particularly in the pharmaceutical industry [11,12,13].

Almeida and Monte [14] had previously studied three isomers of methyl aminobenzoate, with the ortho isomer showing structural similarity to the MMA-3 analyzed in this study. From the determination of the thermophysical parameters of those aminobenzoates, the authors were able to estimate the enthalpy of the N-H···O intermolecular hydrogen bond in the crystalline phase of the *p*-aminobenzoate of methyl. Their findings revealed that the intermolecular bond had a value of 18.9 kJ·mol^−1^.

In this work, energetic studies were performed using calorimetric and effusion techniques to determine the thermodynamic properties of the two anthranilate derivatives. These studies included the determination of (*a*) the standard (*p*° = 0.1 MPa) molar enthalpy of formation in the condensed phase through static-bomb combustion calorimetry experiments; (*b*) the fusion temperature and enthalpy of fusion through differential scanning calorimetry; (*c*) the vapor pressures at different temperatures using the Knudsen effusion method and calculation of the sublimation enthalpy using the Clapeyron equation; and (*d*) the enthalpies of sublimation and vaporization through high-temperature microcalorimetry. The combination of these parameters allowed for the derivation of the standard molar enthalpies of formation for both molecules in the gaseous phase at *T* = 298.15 K. In addition, computational studies were conducted using the G3(MP2)//B3LYP composite method to estimate the gas-phase standard molar enthalpy of the formation of these substances.

The gaseous phase is often considered a reference state in thermodynamics when studying the relationships involving structure, energy, and reactivity of molecules. In the gaseous phase, molecules are far apart from each other, so the predominant interactions are intramolecular bonds. This means that the interactions among molecules, such as intermolecular forces, may be considered negligible. By focusing on the gaseous phase, it is possible to isolate the contribution of intramolecular interactions. This simplification allows for a more straightforward analysis of the relationship between the molecular structure and the thermodynamic properties of a compound. One such thermodynamic parameter that is often used in this context is the standard molar enthalpy of formation in the gaseous state, ${∆}_{f}H_{m}^{{}^{\circ}}(g)$. By knowing the values of this parameter for a set of structurally similar molecules, it becomes possible to calculate the enthalpy increments, $∆∆H_{m}^{{}^{\circ}}(g)$, associated with their structural differences. These enthalpy increments can provide insights into how different structural features, such as functional groups or substituents, affect the overall energy of the molecule and its reactivity. They can also be used to compare the stability or energy differences between the isomers or analogs and help predict how these differences might influence chemical reactions and physical properties.

## 2. Results

### 2.1. Computational Studies

Computational thermochemistry was used to study the thermodynamic properties of the four methyl methylanthranilate (MMA) isomers in the gas phase: MMA-3, MMA-4, MMA-5, and MMA-6. The G3(MP2)//B3LYP composite method [15] was employed for this purpose, as it has exhibited exceptional performance in previous studies [9,16,17]. A conformational analysis was developed to identify and quantify the various stable conformations for each isomer of MMAs. Additionally, the gas-phase enthalpy of formation, ${∆}_{f}H_{m}^{{}^{\circ}}\left(g\right)$, was estimated for each isomer, taking into consideration the composition of conformers.

#### 2.1.1. Conformational Composition

**Figure 2:**
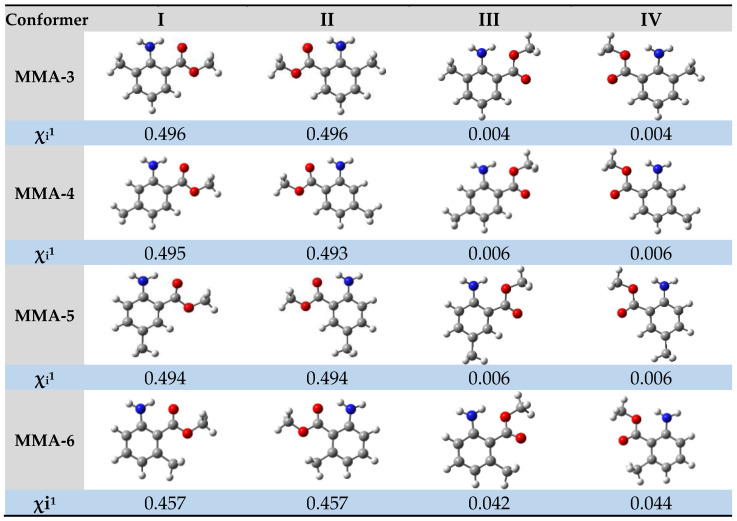
Conformational composition, χ_i_, for the most stable predominant molecular geometries obtained by the G3(MP2)//B3LYP composite method for different isomers of methyl methylanthranilate. Atom color code: grey—C; red—O; white—H; blue—N. ^1^ Conformer composition, χ_i_, determined from Boltzmann distribution.

Due to the different possible arrangements of each substituent in the gaseous phase of MMA isomers, a comprehensive conformational analysis was conducted to determine the most stable conformers. Four optimized low-energy structures were identified (without imaginary frequencies) for each isomer, as shown in Figure 2.

The conformational composition, χ_i_, indicated in the figure for each of the conformers, was calculated assuming a Boltzmann distribution of the *n* possible equilibrium structures. The conformers for each isomer can be divided into two sets, primarily differing in the orientation of the ester group. Additionally, within each set, it is observed that the hydrogens of the amino group extend beyond the plane of the benzene ring due to the sp^3^ hybridization of the nitrogen atom. As a result, these conformers exhibit mirror-image relationships, making them non-superimposable to one another. Other conformations of methyl anthranilate isomers were found with a residual probability, so they were not considered for the final conformational composition. The details of the conformational analysis performed are provided in Tables S1–S4 in Supplementary Materials.

#### 2.1.2. Gas-Phase Standard Molar Enthalpy of Formation

The theoretical values of the ${∆}_{f}H_{m}^{{}^{\circ}}\left(g\right)$ for MMA isomers were estimated considering gas-phase hypothetical reactions, mainly atomization and isodesmic reactions. In this process, the enthalpy change of those reactions, ${∆}_{r}H_{m}^{{}^{\circ}}\left(g\right)$, were calculated using the computational absolute standard enthalpies, $H_{298.15K}^{{}^{\circ}}$, of the structures involved (considering the conformation composition of each one) through Equation (1). Then, with the knowledge of experimental gas-phase standard molar enthalpies of formation of the auxiliary molecules involved in the gas-phase hypothetical reactions, the estimated values of ${∆}_{f}H_{m}^{{}^{\circ}}\left(g\right)$ were calculated using Equation (2). The data pertaining to these chemical structures are presented in Supplementary Materials (Table S5).

(1) $${∆}_{r}H_{m}^{{}^{\circ}}\left(g\right)=\Sigma H_{298.15K}^{{}^{\circ}}\;(g,\;\mathrm{products})-\Sigma \;H_{298.15K}^{{}^{\circ}}(g,reagents)$$

(2) $${∆}_{r}H_{m}^{{}^{\circ}}\left(g\right)=\Sigma {∆}_{f}H_{m}^{{}^{\circ}}\;(g,\;\mathrm{products})-\Sigma {∆}_{f}H_{m}^{{}^{\circ}}(g,reagents)$$

In Table 1, the hypothetical gas-phase reactions proposed are reported, along with their corresponding values of the ${∆}_{f}H_{m}^{\circ }\left(g\right)$ for the four MMA isomers. The range of values obtained for each isomer remains within 11 kJ·mol^−1^, leading to the inclusion of all values in calculating the average. These values, along with estimated entropic effects in the gas phase, enabled the computation of gas-phase Gibbs energy formation, ${∆}_{f}G_{m}^{\circ }\left(g\right)$, for each isomer, with values detailed in the final row of Table 1. 

### 2.2. Experimental Studies

Throughout this experimental study, calorimetric and Knudsen effusion techniques were employed, enabling the determination of the thermodynamic properties of MMA-3 and MMA-5.

#### 2.2.1. Enthalpies of Combustion and Formation in the Condensed Phase

Combustion experiments were conducted for the two anthranilate isomers using static-bomb combustion calorimetry in oxygen [18]. The general combustion reaction for MMA-3 (l) and MMA-5 (cr) is illustrated in Equation (3).

(3) $$C_{9}H_{11}NO_{2}\;(\mathrm{cr},l)+10.75O_{2}\;(g)\;\to \;9\;{\mathrm{CO}}_{2}\left(g\right)+5.5\;H_{2}O\;(l)+0.5\;N_{2}\;(g)$$

The internal energy associated with the isothermal bomb process, $∆U\left(\mathrm{IBP}\right)$, was calculated using Equation (4), where ${∆T}_{\mathrm{ad}}$ represents the calorimeter temperature change corrected for heat exchange and stirring work, $∆m\left(H_{2}O\right)$ is the difference between the mass of water added to the calorimeter and the mass of 3119.6 g assigned to $\varepsilon \left(\mathrm{cal}\right)$, $\varepsilon_{f}$ is the energy equivalent of the content in the final state, ∆U(ign) is the electric energy required for ignition, and $c_{p}\left(H_{2}O,\;l\right)$ is the specific heat capacity at constant pressure for liquid water.

(4) $$∆U\left(\mathrm{IBP}\right)=-\left\{\varepsilon \left(\mathrm{cal}\right)+∆m\left(H_{2}O\right)\cdot c_{p}\left(H_{2}O,\;l\right)+\varepsilon_{f}\right\}{∆T}_{\mathrm{ad}}+∆U\left(\mathrm{ign}\right)$$

The method described by Hubbard et al. [19] was used to calculate the standard massic energy of combustion, ${∆}_{c}u^{{}^{\circ}}$ and the corrections to the standard state, ${∆U}_{\Sigma }$, for combustions occurring in a bomb calorimeter at constant volume. Additionally, several terms must be considered to properly account for the energy associated with side reactions. These terms include the energy of standard formation of nitric acid solution, the energy of combustion of cotton fuse, the energy of combustion of carbon formed by incomplete combustion, the combustion energy of Melinex (used as a combustion aid), and the ignition energy. Detailed combustion results for the two MMA isomers can be found in Tables S6 and S7, which are included in Supplementary Materials.

**Table 1:** Hypothetical gas-phase reactions for the theoretical study of the MMA isomers and corresponding calculated values for the gas-phase enthalpy of formation, ${∆}_{f}H_{m}^{\circ }(g)$, and gas-phase Gibbs energy of formation, , and gas-phase Gibbs energy of formation, ${∆}_{f}G_{m}^{\circ }(g)$, at *T* = 298.15 K ^1^.

Hypothetical Gas-Phase Reactions		${∆}_{f}H_{m}^{\text{∘}}\left(g\right)$/kJ·mol^−1^			
		MMA-3 X_1_=CH_3_, X_2_=X_3_=X_4_=H	MMA-4 X_2_=CH_3_, X_1_=X_3_=X_4_=H	MMA-5 X_3_=CH_3_, X_1_=X_2_=X_4_=H	MMA-6 X_4_=CH_3_, X_1_=X_2_=X_3_=H
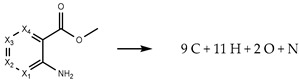	H1	−323.08	−322.58	−318.53	−303.46
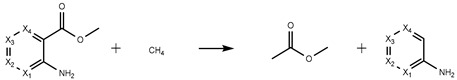	H2	−323.84	−325.57	−315.08	−306.49
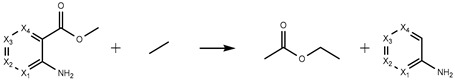	H3	−321.21	−322.95	−312.45	−303.86
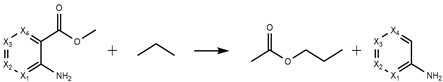	H4	−325.53	−327.26	−316.77	−308.18
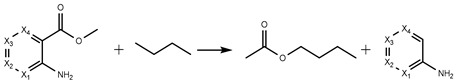	H5	−321.70	−323.43	−312.93	−304.35
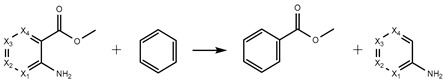	H6	−316.12	−317.85	−307.35	−298.77
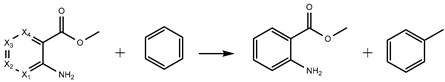	H7	−319.14	−318.64	−314.58	−299.51
$<{∆}_{f}H_{m}^{\text{∘}}\left(g\right)>$ (considering the conformer composition)^2^/kJ·mol^−1^		−321.5 ± 2.9	−322.6 ± 3.2	−314.0 ± 3.3	−303.5 ± 3.2
${∆}_{f}G_{m}^{\text{∘}}\left(g\right)$/kJ·mol^−1^		−115	−128.5	−132.1	−137.3

^1^ The G3(MP2)//B3LYP absolute enthalpies and the experimental gas-phase enthalpies of formation of all the auxiliary compounds are reported in Table S5 of Supplementary Materials [9,20,21,22,23,24,25]; ^2^ The uncertainty assigned corresponds to the expanded uncertainty determined from the estimated standard deviation of the mean for seven reactions (H1–H7) and the coverage factor *k* = 2.5 (0.95 level of confidence and six degrees of freedom) [26].

The mean values obtained for the standard molar combustion energies, ${∆}_{c}u^{{}^{\circ}}$, in the condensed phase of MMA-3 and MMA-5 from six combustion experiments were (−28569.6 ± 12.6) J·g^−1^ and (−28460.9 ± 8.1) J·g^−1^, respectively. The quoted uncertainty corresponds to the standard deviation of the mean. The standard molar values for the internal energy, ${∆}_{c}U_{m}^{{}^{\circ}}(\mathrm{cr},l)$, and enthalpy, ${∆}_{c}H_{m}^{{}^{\circ}}(\mathrm{cr},l)$, corresponding to the combustion reaction Equation (3) are listed in Table 2 for each of the compounds. The standard molar enthalpies of formation, ${∆}_{f}H_{m}^{{}^{\circ}}(\mathrm{cr},l)$, of each MMA isomer at *T* = 298.15 K were derived from the value of ${∆}_{c}H_{m}^{{}^{\circ}}(\mathrm{cr},l)$ and from the following values of ${∆}_{f}H_{m}^{{}^{\circ}}$ of the species involved in the combustion at *T* = 298.15 K: (−285.830 ± 0.042) kJ·mol^−1^ [27] for H_2_O (l) and (−393.51 ± 0.13) kJ·mol^−1^ [27] for CO_2_ (g).

#### 2.2.2. Temperature and Enthalpy of Fusion by DSC

The thermal behavior study of the MMA-5 crystal was conducted using differential scanning calorimetry (DSC) within the temperature range of 298.15 K to 358.15 K.

Table 3 presents both the individual values obtained and the average value for the six experiments conducted to determine the fusion temperature, *T*_f_, and enthalpy of fusion ${∆}_{cr}^{l}H_{m}^{{}^{\circ}}(T_{f})$ at temperature of fusion. The *T*_f_ corresponding to the onset temperature was found to be (335.31 ± 0.04) K. Prior to the crystal–liquid transition, no crystal–crystal transitions were observed, as it is evident from the DSC curve provided in Supplementary Materials. In addition to this, the ${∆}_{cr}^{l}H_{m}^{{}^{\circ}}(T_{f})$ was determined by calculating the area under the peak observed in the DSC curve corresponding to the crystal-to-liquid transition. The area is converted to heat by using the energy calibration of the DSC instrument. Assuming a constant thermal expansion coefficient of the sample, the enthalpy of fusion for the compound can be calculated at the fusion temperature, with a mean value of (22.0 ± 0.3) kJ·mol^−1^.

#### 2.2.3. Enthalpies of Sublimation and Vaporization by Calvet Microcalorimetry

The techniques for measuring the enthalpies of phase transition from condensed to gaseous phases for the compounds under study were chosen based on the physical state of the samples at room temperature. Therefore, the enthalpy of sublimation of the crystal MMA-5 was measured by Calvet microcalorimetry and the Knudsen effusion method, while the enthalpy of vaporization of the liquid MMA-3 was measured by Calvet microcalorimetry.

In the Calvet microcalorimeter, the study of each compound required a different temperature of the block calorimeter: 345.6 K for MMA-3 and 333.6 K for MMA-5. At each of these temperatures, an in situ calibration was performed using appropriate reference substances. Specifically, decane was used for the temperature of 345.6 K, and naphthalene was used for the temperature of 333.6 K. Table 4 shows the summary of results obtained for the isomers (the overall results are given in Tables S8 and S9 of Supplementary Materials).

The observed enthalpy of phase transition at the experimental temperature *T*, ${∆}_{\mathrm{cr},l,298.15K}^{g,T}H_{m}^{{}^{\circ}}$, for each compound, corresponds to the mean of the results of six experiments. The molar heat capacities in the gaseous phase for each compound were used to adjust these values to *T* = 298.15 K using eqs. 5 and 6. The molar heat capacities in the gaseous phase for both compounds were determined through statistical thermodynamics, which used the vibrational frequencies resulting from B3LYP calculations with the 6-31G(*d*) basis set. These vibrational frequencies were scaled by a factor of 0.960 ± 0.022 [28]. The gas-phase molar heat capacities at various temperatures and the resulting mathematical expressions in the form of polynomial functions for each compound can be found in the supplementary data provided in Supplementary Materials (Table S10 and Equations (S1) and (S2)).

(5) $${∆}_{298.15K}^{T}H_{m}^{o}\left(g\right)=\int_{298.15K}^{T}C_{p}^{{}^{\circ}}\left(g\right)dT$$

(6) $${∆}_{\mathrm{cr},l}^{g}H_{m}^{{}^{\circ}}={∆}_{\mathrm{cr},l,\;298.15K}^{g,\;T_{}}H_{m}^{{}^{\circ}}-\;{∆}_{298.15\;K}^{T_{}}H_{m}^{{}^{\circ}}(g)$$

The standard molar enthalpies of phase transition, at *T* = 298.15 K, derived were: ${∆}_{l}^{g}H_{m}^{{}^{\circ}}$ = (70.8 ± 2.0) kJ·mol^−1^ for MMA-3 and ${∆}_{cr}^{g}H_{m}^{{}^{\circ}}$ = (89.9 ± 2.0) kJ·mol^−1^ for MMA-5.

#### 2.2.4. Vapor Pressures and Enthalpies of Sublimation by Knudsen Effusion Method

The measurements of the vapor pressures of the MMA-5 crystalline compound were taken over a temperature range of [293.40–309.09] K, specifically chosen to correspond to the measured vapor pressure range of 0.1 to 1.0 Pa. The results of the Knudsen effusion experiments for the three cell sets (small, S; medium, M; and large, L) are provided in Supplementary Materials. Table 5 displays the Clausius–Clapeyron equation parameters (*a* is a constant that accounts (Table S11).

The Clausius–Clapeyron Equation (7) was used to determine the enthalpy of sublimation of MMA-5 from its vapor pressure data at different temperatures. The plots of ln(*p*/Pa) against 1/*T* for each set of orifices for MMA-5 are shown in Supplementary Materials. Table 5 displays the Clausius–Clapeyron equation parameters (*a* is a constant that accounts for the volume change upon sublimation and *b* is the enthalpy of sublimation at that temperature, divided by the ideal gas constant*)* obtained from the least squares adjustment, equilibrium pressure at the mean temperature, and the standard (*p*° = 0.1 MPa) molar enthalpies of sublimation at the mean temperature of the experiments for each set of effusion cells (S, M, and L) as well as the global results. As can be seen, the calculated enthalpies of sublimation for the different orifices agree with the experimental error. This leads us to conclude that the vapor pressure experiments were conducted under equilibrium conditions, with no deviations observed during the experiments.

(7) $$\ln\left(p/Pa\right)=a-b(K/T)$$

The values for the derived standard molar enthalpy, entropy, and Gibbs energy of sublimation at *T* = 298.15 K are reported in Table 6. More details about the calculations made to obtain these parameters can be found in Supplementary Materials. Table 5 displays the Clausius–Clapeyron equation parameters (*a* is a constant that accounts).

#### 2.2.5. Calculation of Phase Transition Enthalpies for the MMA-5 Isomer

As previously reported, the enthalpy of sublimation for the MMA-5 isomer was determined using Knudsen effusion and Calvet microcalorimetry techniques, resulting in values of (93.4 ± 0.8) kJ·mol^−1^ and (89.9 ± 2.0) kJ·mol^−1^, respectively. When comparing the results obtained from both techniques, a significant level of agreement is observed, considering the corresponding uncertainties. Therefore, it is proposed that the average value of these measurements, (92.9 ± 0.7) kJ·mol^−1^, be considered the sublimation enthalpy of the MMA-5 isomer, considering the respective weight of uncertainty for each technique.

The enthalpy of fusion at the temperature of fusion of MMA-5 obtained from DSC measurements (Table 3) can be adjusted to 298.15 K using the enthalpy changes associated with the difference of temperature in the crystalline and liquid phases, ${∆}_{298.15K}^{T_{\mathrm{fusion}}}H_{m}^{{}^{\circ}}\left(\mathrm{cr}\right)$ and ${∆}_{298.15K}^{T_{\mathrm{fusion}}}H_{m}^{{}^{\circ}}\left(l\right)$, respectively, by means of Equation (8). The resulting value was determined to be (20.2 ± 0.3) kJ·mol^−1^.

(8) $${∆}_{cr}^{l}H_{m}^{{}^{\circ}}(298.15K)={∆}_{cr}^{l}H_{m}^{{}^{\circ}}(T_{\mathrm{fusion}})+{∆}_{298.15K}^{T_{\mathrm{fusion}}}H_{m}^{{}^{\circ}}\left(\mathrm{cr}\right)-{∆}_{298.15K}^{T_{\mathrm{fusion}}}H_{m}^{{}^{\circ}}\left(l\right)$$

The enthalpy changes associated with the difference in temperature in the crystalline and liquid phases, as indicated in the previous equation, respectively, ${∆}_{298.15\;K}^{T_{\mathrm{fusion}}}H_{m}^{{}^{\circ}}\left(\mathrm{cr}\right)$ and ${∆}_{298.15\;K}^{T_{\mathrm{fusion}}}H_{m}^{{}^{\circ}}\left(l\right)$, were calculated by integrating the heat capacities of each phase with respect to temperature. The expressions for the temperature-dependent molar heat capacities in the crystalline Equation (9) and liquid phases Equation (10) for MMA-5 were derived from the equations proposed by Chickos et al. [29], taking into account the gas-phase heat capacity at 298.15 K determined by theoretical calculations.

(9) $$C_{p}^{{}^{\circ}}\left(\mathrm{cr},298.15K\right)=0.88+1.18\;C_{p}^{{}^{\circ}}\left(g,298.15K\;\right)$$

(10) $$C_{p}^{{}^{\circ}}\left(l,298.15K\;\right)=14.30+1.35\;C_{p}^{{}^{\circ}}\left(g,298.15K\;\right)$$

Additionally, the enthalpy of vaporization for the MMA-5 isomer was obtained by subtracting the enthalpy of fusion from the enthalpy of sublimation, both at *T* = 298.15 K. The resulting value obtained was (72.7 ± 0.8) kJ·mol^−1^.

#### 2.2.6. Gas-Phase Enthalpy of Formation Determined Experimentally

Using the principle of Hess’s Law, it becomes feasible to determine the enthalpy of formation in the gas phase by employing experimental data, such as the enthalpy of formation in the condensed phase (with values in Section 2.2.1.) and the enthalpy of phase transition (with values in Section 2.2.3, Section 2.2.4 and Section 2.2.5). In this context, the enthalpy of formation in the gaseous state was calculated for two compounds, MMA-3 and MMA-5. For MMA-3, the value was determined to be (−325.6 ± 3.2) kJ·mol^−1^, considering its enthalpy of formation in the liquid state (−396.4 ± 2.5) kJ·mol^−1^ and its enthalpy of vaporization (70.8 ± 2.0) kJ·mol^−1^. Similarly, for the isomer MMA-5, the enthalpy of formation in the gaseous state was found to be (−314.8 ± 3.0) kJ·mol^−1^, taking into account its enthalpies of formation in the crystalline state (−407.7 ± 2.9) kJ·mol^−1^ and of sublimation (92.9 ± 0.7) kJ·mol^−1^.

## 3. Discussion

As mentioned in earlier sections of this article, the gas-phase enthalpy of formation for the isomers MMA-3 and MMA-5 was assessed using both experimental and computational methods, and the findings are compiled in Table 7. These isomers show a remarkable agreement between the experimental and computational values, with differences lower than ≈ 4 kJ·mol^−1^. This strong alignment suggests that the theoretical method used accurately approximates the real properties of the chemical system under investigation. Such validation of the theoretical approach boosts confidence in the obtained results and indicates that it can be reliably applied to predict the gas-phase enthalpy of formation for similar molecules, including the remaining isomers.

**Figure 3:**
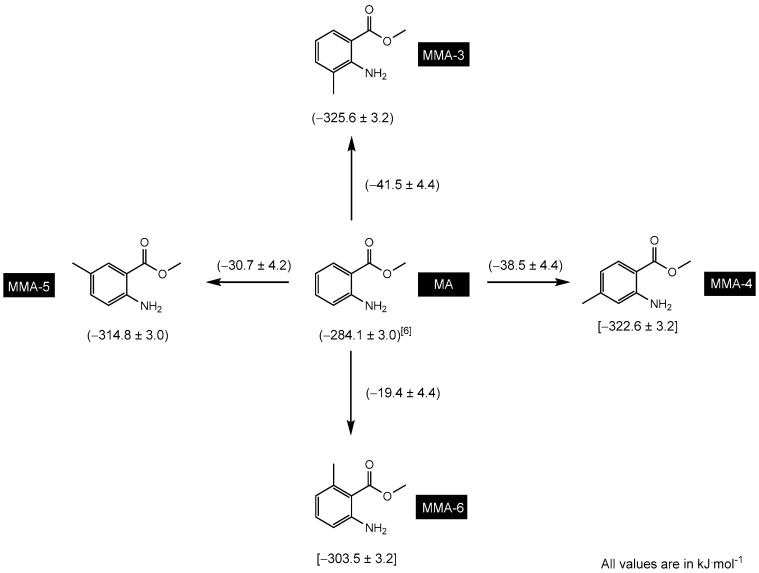
Increment scheme to assess the substitution of a hydrogen atom in the benzene ring with a methyl group at positions 3–6 of the methyl anthranilate (MA) molecule. The values enclosed in square brackets represent values determined using theoretical methods.

One of the objectives of this study was to determine the enthalpic increments, $∆∆H_{m}^{{}^{\circ}}(g)$, associated with structural variations among the molecules being examined. To accomplish this, Figure 3 provides an increment scheme that focuses on assessing the substitution of a hydrogen atom in the benzene ring with a methyl group at positions 3–6 of the methyl anthranilate molecule. Based on the results obtained in this figure, it is evident that the enthalpic increment falls within the range of −42 to −19 kJ·mol^−1^, depending on the position of the methyl group. Previous studies have also provided evidence supporting a similar order of magnitude for the $∆∆H_{m}^{{}^{\circ}}(g)$ in fundamental molecules like benzofuran derivatives, benzothiophene, dibenzofuran, and dibenzothiophene [30,31]. This consistency leads to the consideration of an average value for the hydrogen-to-methyl replacement increment, estimated to be approximately −30 kJ·mol^−1^.

**Figure 4:**
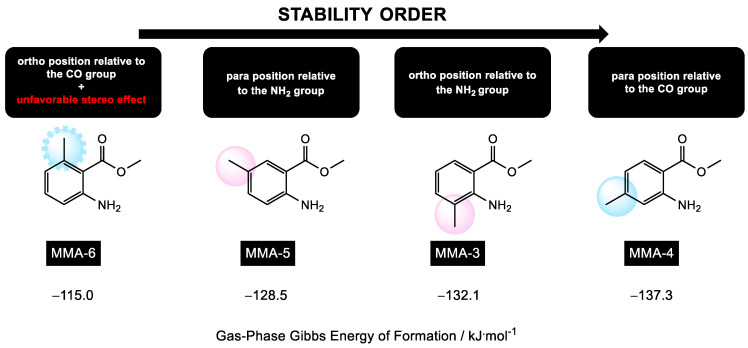
Schematic representation of the order of stability of MMA isomers, considering the values of the gas-phase Gibbs energy of formation, estimated by the theoretical method used in this research.

Figure 4 displays the gas-phase Gibbs energy values of formation, ${∆}_{f}G_{m}^{{}^{\circ}}(g)$, for each of the isomers of methyl methylanthranilate estimated by the theoretical method used in this study. Based on these values, the isomers can be arranged in the following order of increasing stability: MMA-6, MMA-5, MMA-3, and MMA-4. This stability trend can be potentially explained by considering the inductive and resonant effects of the methyl group in different positions, as well as possible stereo effects caused by this one.

The methyl substituent can have an electron-donating effect through both inductive and resonant effects, thereby increasing the electron density in a molecule. This effect is particularly significant when the methyl group is in an ortho or para position relative to electronically active functional groups, such as carbonyl and amine groups. The inductive effect of the methyl group leads to an increase in electron density in the molecule, resulting in a more negative carbonyl carbon and a more polarized carbon–oxygen bond (C=O). Additionally, it may cause the amino nitrogen atom to become more polarized, making the amine group more negative and, thus, more nucleophilic.

The resonant effect is generally more potent than the inductive effect when it comes to electron donation by a methyl group or any other functional group. This is because the resonant effect involves the delocalization of electrons through multiple π bonds or lone pairs, leading to greater electronic delocalization and molecular stabilization [32]. On the other hand, the inductive effect involves only a partial charge redistribution across a σ bond, resulting in less electronic influence compared to resonance. Both effects, the inductive and the resonant, act cooperatively to increase the stability of the system and influence the chemical and reactive properties of the amine group and the carbonyl group in the molecule. It is important to emphasize that the specific nature of these effects will depend on the exact structure of the molecule and the interactions between the functional groups.

As previously mentioned, the MMA-6 isomer is the least stable. The unfavorable steric interaction between the methyl and carbonyl groups, caused by their arrangement and proximity, seems to override the inductive and resonant effects. In contrast, the MMA-5 isomer is more stable due to the favorable position of the methyl group relative to the amine and carbonyl groups. This arrangement minimizes steric interactions and favors the electron-donating effects of the methyl group. Next, the MMA-3 isomer is considered, where the methyl group is in an ortho position relative to the amine group. This positioning contributes to the molecule’s stabilization through inductive and resonant effects, and the steric interaction between the two substituents is not significant. The MMA-4 isomer is the most stable among the MMA isomers. The methyl group is in a para-position relative to the carbonyl group, leading to greater effectiveness of the electron-donating effect. Steric interactions are minimized both for the amine and carbonyl groups, contributing to the overall stability of the molecule.

## 4. Materials and Methods

### 4.1. Materials and Purification Processes

Both anthranilate derivatives, methyl 3-methylanthranilate, MMA-3 (CAS Registry No. 22223-49-0), and methyl 5-methylanthranilate, MMA-5 (CAS Registry No. 18595-16-9), were commercially acquired from TCI^®^ (Deutschland GmbH, Eschborn, Germany) with molar fractions exceeding 0.99. The liquid sample of MMA-3 underwent purification through distillation under reduced pressure, achieving a final purity of 0.9987. The purity analysis of the commercial sample MMA-5 by gas-liquid chromatography showed no impurities, making additional purification methods unnecessary. Samples of the two MMA isomers studied experimentally are summarized in Table 8.

### 4.2. Combustion Calorimetry

The enthalpies of combustion for the two fragrance compounds were determined using an isoperibol static-bomb combustion calorimetry setup, equipped with a twin-valve 1108 Parr Instrument Company bomb with an internal volume of 0.342 dm^3^. A detailed description of the calorimetric system can be found in the literature [18].

In sample preparation, the MMA-3 liquid samples were placed inside polyester bags made of Melinex^®^, following the procedure described by Skinner and Snelson [33]. This step prevents the vaporization of the sample and any subsequent loss of mass. For the crystalline sample of MMA-5, there was no need to use any combustion aids. During the bomb assembly, the sample (with or without combustion aid) was introduced into a platinum crucible with a cotton thread fuse attached to the platinum ignition wire (Goodfellows, diameter = 0.05 mm). Additionally, 1.00 cm^3^ of deionized water was added to the bottom of the bomb to maintain a vapor phase saturated with H_2_O throughout the experiment. The bomb was sealed, and prior to filling it with oxygen ($x_{O_{2}}$ ≥ 0.99995) at a pressure of 3.04 MPa, it was purged to remove any air. Subsequently, the bomb was placed inside the calorimeter along with 3119.6 g of distilled water (calorimetric liquid). The temperature of the water was continuously measured and monitored over time using a Hewlett–Packard 2804A quartz crystal thermometer with an uncertainty of ±(1∙10^−4^) K, which was interfaced to a computer setup running the LABTERMO program to calculate the adiabatic temperature [34]. The ignition of the sample occurred at *T* = (298.1500 ± 0.0001) K, achieved by discharging a 1400 μF capacitor through the platinum ignition wire. Prior to ignition, 100 temperature readings were recorded, followed by 200 readings after ignition. The thermostatic bath was maintained at approximately 301.15 K using Tronac, a temperature controller.

During the combustion experiments, gravimetric analysis was used to collect carbon dioxide, while acid–base titration was employed to quantify the nitric acid. The mass of carbon dioxide produced directly corresponds to the amount of compound burned in each experiment.

The energy equivalent of the calorimeter was determined using the combustion of benzoic acid in standard reference material (SRM) 39j, provided by the National Institute of Standards and Technology [35]. This certified material has a massic energy of combustion of (−26434 ± 3) J·g^−1^. Calibration experiments were conducted under the same conditions as the compound experiments. The energy equivalent of the calorimeter, ε(cal), was calculated to be (16,002.6 ± 1.7) J·K^−1^, based on an average mass of water added to the calorimeter of 3119.6 g. The quoted uncertainty represents the standard deviation of the mean for six experiments.

The compounds and combustion aids were weighed using a Mettler AE240 balance with a precision of ± (1·10^−5^) g, while the mass of water added to the calorimetric vessel was measured using a Mettler PC 8000 balance with a sensitivity of ±(1·10^−1^) g.

### 4.3. High-Temperature Microcalorimetric Technique

The molar phase transition enthalpies at standard pressure (*p*° = 0.1 MPa), ${∆}_{\mathrm{cr},l}^{g}H_{m}^{{}^{\circ}}$, were determined by a high-temperature Calvet microcalorimeter, model HT1000, provided by Setaram. Detailed information about the apparatus and the experimental technique can be found in the literature [36].

Samples weighing between 4 and 7 mg were placed inside thin glass capillary tubes, with one end sealed. These capillary tubes, along with an empty one, were then dropped into the block calorimeter, programmed at temperatures of approximately 345.6 K and 333.6 K for MMA-3 and MMA-5, respectively, starting from room temperature. Once thermodynamic stability was achieved, the phase transition of the samples occurred under vacuum conditions. Data acquisition of the heat flux versus time and subsequent analysis were performed using an interfaced computer. The integration of the resulting thermogram yields the observed standard molar phase transition enthalpy, ${∆}_{\mathrm{cr},l,298.15K}^{g,T}H_{m}^{{}^{\circ}}$, which is then corrected to *T* = 298.15 K, ${∆}_{\mathrm{cr},l}^{g}H_{m\;}^{{}^{\circ}}$**.**

The microcalorimeter was calibrated in situ at both temperatures by determining the phase transition enthalpies of reference materials, such as decane with ${∆}_{l}^{g}H_{m}^{{}^{\circ}}$ = (51.4 ± 0.2) kJ·mol^−1^ [37], and naphthalene with ${∆}_{\mathrm{cr}}^{g}H_{m}^{{}^{\circ}}$ = (72.6 ± 0.6) kJ·mol-1 [37], for vaporization and sublimation enthalpies, respectively, using the same experimental procedure. The calibration constants, k_cal_, were determined for each of the compounds under the experimental conditions, resulting in values of (1.0289 ± 0.0139) and (1.0565 ± 0.0034) for MMA-3 and MMA-5, respectively. The uncertainty represents the estimated standard deviation of the mean for six and seven experiments, respectively.

The mass measurements of the samples and capillary tubes were carried out with a precision of ± (1 ± 10^−7^) g using a Mettler–Toledo UMT2 microbalance.

### 4.4. Knudsen Mass-Loss Effusion Method

The standard molar enthalpy of sublimation, ${∆}_{\mathrm{cr}}^{g}H_{m}^{{}^{\circ}}$ for MMA-5, was determined using the Knudsen mass-loss effusion method. Vapor pressures were measured over a range of 0.1 to 1 Pa using an apparatus equipped with three aluminum blocks. Each block could handle three effusion cells, allowing nine effusion cells to be operated simultaneously at three different temperatures. Details about the apparatus and the experimental technique can be found in the literature [38].

For the experiment, samples were introduced and sealed in nine aluminum effusion cells, which were then placed in cylindrical cavities within the three oven blocks located in a vacuum chamber. The cells in each block were grouped based on three different orifice sizes: small, medium, and large, with orifice areas of approximately 0.64 mm^2^, 0.79 mm^2^, and 0.99 mm^2^, respectively. The effusion orifices were made from a platinum foil with a thickness of 0.0125 mm. Each block maintained a constant temperature.

Vapor pressures, *p*, within the described range were calculated using Equation (11), based on the mass variation of the cells (Δ*m*) and orifice sizes. In this equation, A_0_ represents the area of the effusion orifice, ω_0_ is the transmission probability factor (also known as the Clausing factor) calculated by Equation (12), *t* is the effusion period, *R* is the gas constant (*R* = 8.314472 J∙K^−1^∙mol^−1^), *T* is the selected temperature, and *M* is the molar mass of the effusing vapor. In Equation (12), *l* is the thickness of the platinum foil, and r is the radius of each orifice.

(11) p=mA0w0t·2πRTM12

(12) $$\omega_{0}={\left[1+\left(l/8r\right)\right]}^{-1}$$

The mass measurements of the samples and effusion cells were performed with a precision of ± (1·10^−5^) g using a Mettler AE163 balance.

### 4.5. Computational Methods

Theoretical calculations of the molecular structures were performed using the Gaussian-09 software [39]. These calculations were based on a variation of the Gaussian-3 (G3) theory [40], specifically the composite method G3(MP2)//B3LYP [15].

The enthalpy of formation for both MMA-3 and MMA-5 at *T* = 298.15 K was estimated using gas-phase work reactions, which involved atomization and hypothetical gas-phase group substitution reactions.

## 5. Conclusions

The thermodynamic values obtained in this study have addressed the lack of available data in the literature for this particular class of compounds. This contribution also enhances the potential for more reliable future prediction schemes in evaluating the environmental risks associated with the use of these compounds.

Remarkably, a significant agreement is found between the experimental and computational values for the enthalpy of formation in the gaseous state concerning the MMA-3 and MMA-5 isomers. This agreement validates the computational methodology employed, consequently enabling a reliable prediction of values for the MMA-4 and MMA-6 isomers, for which experimental determination was unfeasible.

The hydrogen-to-methyl replacement enthalpic increment is estimated to be around -30 kJ·mol^−1^.

The observed decreased stability of the MMA-6 isomer might be attributed to a structural concern, possibly stemming from steric strain due to the close proximity of the methyl substituent group to the ester group.

## Figures and Tables

**Table 2:** Standard molar values for methyl 3-methylanthranilate (MMA-3) and methyl 5-methylanthranilate (MMA-5) at *T* = 298.15 K and *p*° = 0.1 MPa in the condensed phase obtained from combustion calorimetry experiments.

Compound	${∆}_{c}U_{m}^{º}$/kJ·mol^−1^	${∆}_{c}H_{m}^{º}$/kJ·mol^−1^	${∆}_{f}H_{m}^{º}$/kJ·mol^−1^
MMA-3 (l)	−4714.1 ± 2.2 ^1^	−4717.2 ± 2.2 ^1^	−396.4 ± 2.5 ^2^
MMA-5 (cr)	−4702.9± 2.6 ^1^	−4705.9 ± 2.6 ^1^	−407.7 ± 2.9 ^2^

^1^ The quoted uncertainty was determined by considering the combined standard uncertainty, which includes the contribution from the calibration with benzoic acid and the auxiliary compound (when used), as well as the coverage factor k = 2 (corresponding to a 0.95 confidence level) [26]; ^2^ The quoted uncertainty was calculated by considering the combined standard uncertainty, which takes into account the contribution of the species participating in the respective combustion reactions Equation (3), and the coverage factor k = 2 (corresponding to a 0.95 confidence level) [26].

**Table 3:** DSC experimental values for MMA-5 fusion temperature and enthalpy of fusion.

Exp	$T_{f}$/K	${∆}_{cr}^{l}H_{m}^{{}^{\circ}}(T_{f})$/kJ·mol^−1^
1	335.36	22.38
2	335.27	22.04
3	335.31	22.04
4	335.34	22.22
5	335.26	21.60
6	335.31	21.74
Mean value	<335.31 ± 0.04> ^1^	<22.0 ± 0.3> ^2^

^1^ The quoted uncertainty was determined by considering the combined standard uncertainty (which includes the uncertainty associated with the temperature calibration) and a coverage factor of k = 2.5, which corresponds to a 0.95 confidence level [26]; ^2^ The quoted uncertainty was determined by considering the combined standard uncertainty (which includes the uncertainty associated with the energy calibration) and a coverage factor of k = 2.5, which corresponds to a 0.95 confidence level [26].

**Table 4:** Standard (*p*° = 0.1 MPa) molar enthalpies of sublimation and vaporization determined by Calvet microcalorimetry for MMA-3 and MMA-5.

Compound (Phase Transition)	*T*/K	${∆}_{\mathrm{cr},l,298.15K}^{g,T}H_{m}^{{}^{\circ}}$/kJ·mol^−1^	${∆}_{298.15K}^{T}H_{m}^{{}^{\circ}}\left(g\right)$/kJ·mol^−1^	${∆}_{\mathrm{cr},l}^{g}H_{m\;}^{{}^{\circ}}$/kJ·mol^−1^
MMA-3 (l → g)	345.6	80.8 ± 0.1 ^1^	10.0 ± 0.2 ^2^	70.8 ± 2.0 ^3^
MMA-5 (cr → g)	333.6	97.3 ± 0.7 ^1^	7.4 ± 0.2 ^2^	89.9 ± 2.0 ^4^

^1^ The quoted uncertainty corresponds to the estimated standard deviation of the mean for six experiments; ^2^ The quoted uncertainty includes the root-mean-square deviation of the third-degree polynomial used and the uncertainty of the vibrational frequency scaling factor for the B3LYP/6-31G(d) method [26,28]; ^3^ The combined standard uncertainty (which includes the uncertainty associated to calibration experiments with decane) and the coverage factor k = 2.16 (for 0.95 level of confidence with an effective degree of freedom of 13, calculated by the Welch–Satterthwaite formula) were used to determine the quoted uncertainty [26]; ^4^ The combined standard uncertainty (which includes the uncertainty associated to calibration experiments with naphthalene) and the coverage factor k = 2.23 (for 0.95 level of confidence with an effective degrees of freedom of 11, calculated by the Welch-Satterthwaite formula) were used to determine the quoted uncertainty [26].

**Table 5:** Parameters of the Clausius-Clapeyron equation obtained for MMA-5 and calculated values for the pressure and enthalpy of sublimation at a mean temperature of 301.25 K.

Orifices	Clausius-Clapeyron Parameters		*R* ^2^	Calculated Values at <*T*> = 301.25 K	
	*a*	*b*		*p*(<*T*>)/Pa	${∆}_{\mathrm{cr}}^{g}H_{m\;}^{{}^{\circ}}$(<*T*>/kJ∙mol^−1^
S1-S2-S3	36.5 ± 0.4 ^1^	11,253 ± 127 ^1^	0.9997	0.426	93.6 ± 1.1 ^3^
M4-M5-M6	36.5 ± 0.5 ^1^	11,248 ± 153 ^1^	0.9996	0.433	93.5 ± 1.3 ^3^
L7-L8-L9	36.1 ± 0.3 ^1^	11,149 ± 92 ^1^	0.9999	0.403	92.7 ± 0.8 ^3^
All	36.4 ± 0.3 ^2^	11,217 ± 96 ^2^	0.9994	0.434	93.3 ± 0.8 ^3^

^1^ The standard uncertainties were determined by multiplying the standard error of the fitting parameters by k = 2.23, which corresponds to the *t*-distribution value for a 95% confidence level and 10 degrees of freedom [26]; ^2^ The standard uncertainties were determined by multiplying the standard error of the fitting parameters by k = 2.03, which corresponds to the *t*-distribution value for a 95% confidence level and 34 degrees of freedom [26]; ^3^ The quoted uncertainties correspond to the combined standard uncertainties at a 95% confidence level.

**Table 6:** Standard (*p*° = 0.1 MPa) molar enthalpies, entropies, and Gibbs energies of sublimation, as well as the vapor pressure, at *T* = 298.15 K, for MMA-5.

${∆}_{\mathrm{cr}}^{g}H_{m}^{{}^{\circ}}$/kJ·mol^−1^	${∆}_{\mathrm{cr}}^{g}S_{m}^{{}^{\circ}}$/kJ·mol^−1^	${∆}_{\mathrm{cr}}^{g}G_{m}^{{}^{\circ}}$/kJ·mol^−1^	*p*/Pa
93.4 ± 0.8	207.4 ± 2.7	31.6 ± 1.1	2.95 × 10^−1^

**Table 7:** Compilation of enthalpy of formation values in the gaseous state determined by experimental and computational studies, along with the differences obtained for these values.

Compound	${∆}_{f}H_{m}^{{}^{\circ}}$(g)/kJ·mol^−1^		∆∆H(g) ^1^/kJ·mol^−1^
	Experimental	Computational	
**MMA-3**	−325.6 ± 3.2	−321.5 ± 2.9	−4.1
**MMA-5**	−314.8 ± 3.0	−314.0 ± 3.3	−0.8

^1^ The calculation formula for this difference was the subtraction of the value of the experimental gas-phase enthalpy of formation from the computational one.

**Table 8:** Compound details, CAS registry number, supplier, and sample Purity.

Compound [IUPAC Name]	Acronym	CAS Registry No.	Supplier	Initial Purity	Purification Method	Final Mass Fraction Purity
Methyl 3-methylanthranilate [Methyl 2-amino-3-methylbenzoate]	MMA-3	22223-49-0	TCI^®^	0.998 ^1^	Vacuum distillation	0.9987 ^2^
Methyl 5-methylanthranilate [Methyl 2-amino-5-methylbenzoate]	MMA-5	18595-16-9	TCI^®^	1.000 ^1^	—	0.9999 ^2^

^1^ Values stated in the certificate of analysis of the supplier; ^2^ Results were obtained from gas-liquid chromatography using an Agilent 4890 apparatus equipped with an HP-5 column, which is cross-linked and composed of 5% diphenyl and 95% dimethylpolysiloxane (column dimensions: 15 m in length × 0.530 mm internal diameter × 1.5 μm film thickness).

## Data Availability

Not applicable.

## Supplementary Materials

The following supporting information can be downloaded at: https://www.mdpi.com/article/10.3390/molecules28186686/s1, Tables S1–S4. Absolute standard enthalpies, $H_{298.15\;K}^{{}^{\circ}}$, and entropies, $S_{298.15\;K}^{{}^{\circ}}$, obtained by the G3(MP2)//B3LYP composite method and the corresponding derived gas-phase standard molar enthalpies, ${∆}_{f}H_{m}^{{}^{\circ}}\left(g\right)$, entropies, ${∆}_{f}S_{m}^{{}^{\circ}}\left(g\right)$, and Gibbs energy of formation, ${∆}_{f}G_{m}^{{}^{\circ}}\left(g\right)$, at *T* = 298.15 K, as well as the conformational composition, χ_I_, obtained for, respectively, MMA-3, MMA-4, MMA-5, and MMA-6 Table S5. G3(MP2)//B3LYP enthalpies with corresponding gas-phase standard (*p*° = 0.1 MPa) molar enthalpies of formation, at *T* = 298.15 K, for the atomic and molecular species. Tables S6 and S7. Combustion results and standard (*p*° = 0.1 MPa) massic energy of combustion at *T* = 298.15 K for MMA-3 and MMA-5, respectively. Tables S8 and S9. Calvet microcalorimetry results for the processes of vaporization of MMA-3 and sublimation of MMA-5, respectively. Table S10. Standard (*p*° = 0.1 MPa) molar heat capacities in the gaseous phase for MMA-3 and MMA-5, obtained from statistical thermodynamics using the vibrational frequencies calculated at the B3LYP/6-31G(d) level of theory (scaled by a factor of 0.960 ± 0.022). Table S11. Knudsen mass-loss effusion results for MMA-5 at different orifices areas. Figure S1. Plots of ln(*p*/Pa) against 1/*T* for small, medium, and large orifices obtained by the mass-loss Knudsen effusion experiments for MMA-5. Figure S2. DSC curve obtained for MMA-5. References in Supplementary Materials [41,42].

## Sample Availability

Samples of the compounds are available from the authors.
