# Supplementary material for: Determination and Analysis of Thermodynamic Properties of Methyl Methylanthranilate Isomers
Source: Molecules. 2023 Sep 18;28(18):6686. doi: 10.3390/molecules28186686 (PMC10534601; doi:10.3390/molecules28186686)
Supplement: Supplementary file 1 [file molecules-28-06686-s001.zip › molecules-2579806-supplementary.pdf]

Supplementary Materials

Table of Contents

TABLE S1. Absolute standard enthalpies, $H^\circ_{298.15\,\mathrm{K}}$, and entropies, $S^\circ_{298.15\,\mathrm{K}}$, obtained by G3(MP2)//B3LYP composite method for MMA-3 and the corresponding derived gas-phase standard molar enthalpies, $\Delta_f H^\circ_m$(g), entropies, $\Delta_f S^\circ_m$(g), and Gibbs energy of formation, $\Delta_f G^\circ_m$(g), at T = 298.15 K, and the conformational composition, χ_I ……….… A4

Table S2. Absolute standard enthalpies, $H^\circ_{298.15\,\mathrm{K}}$, and entropies, $S^\circ_{298.15\,\mathrm{K}}$, obtained by G3(MP2)//B3LYP composite method for MMA-4 and the corresponding derived gas-phase standard molar enthalpies, $\Delta_f H^\circ_m$(g), entropies, $\Delta_f S^\circ_m$(g), and Gibbs energy of formation, $\Delta_f G^\circ_m$(g), at T = 298.15 K, and the conformational composition, χ_I ………… A4

Table S3 Absolute standard enthalpies, $H^\circ_{298.15\,\mathrm{K}}$, and entropies, $S^\circ_{298.15\,\mathrm{K}}$, obtained by G3(MP2)//B3LYP composite method for MMA-5 and the corresponding derived gas-phase standard molar enthalpies, $\Delta_f H^\circ_m$(g), entropies, $\Delta_f S^\circ_m$(g), and Gibbs energy of formation, $\Delta_f G^\circ_m$(g), at T = 298.15 K, and the conformational composition, χ_I. ………… A5

Table S4. Absolute standard enthalpies, $H^\circ_{298.15\,\mathrm{K}}$, and entropies, $S^\circ_{298.15\,\mathrm{K}}$, obtained by G3(MP2)//B3LYP composite method for MMA-6 and the corresponding derived gas-phase standard molar enthalpies, $\Delta_f H^\circ_m$(g), entropies, $\Delta_f S^\circ_m$(g), and Gibbs energy of formation, $\Delta_f G^\circ_m$(g), at T = 298.15 K, and the conformational composition, χ_I ………… A5

Table S5. G3(MP2)//B3LYP enthalpies with corresponding gas-phase standard (p^o = 0.1 MPa) molar enthalpies of formation, at T = 298.15 K, for the atomic and molecular species. A6

Table S6. Combustion results and standard ($p°$ = 0.1 MPa) massic energy of combustion, at T = 298.15 K, for MMA-3. ……………………………………………………………………….. A8

Table S7. Combustion results and standard ($p°$ = 0.1 MPa) massic energy of combustion, at T = 298.15 K, for MMA-5. ……………………………………………………………………….. A9

Table S8. Calvet microcalorimetry results for the process of vaporization of MMA-3. ………….. A10

Table S9. Calvet microcalorimetry results for the process of sublimation of MMA-5. …………… A11

Table S10. Standard ($p°$ = 0.1 MPa) molar heat capacities in the gaseous phase for MMA-3 and MMA-5, obtained from statistical thermodynamics using the vibrational frequencies calculated at the B3LYP/6-31G(d) level of theory (scaled by a factor of 0.960 ± 0.022). A11

Table S11. Knudsen mass-loss effusion results for MMA-5 at different orifices areas. …………… A12

Figure S1. Plots of ln(p/Pa) against 1/T for small, medium and large orifices obtained by the mass-loss Knudsen effusion experiments for MMA-5. ………………………………….. A14

Acronyms

MMA-3, methyl 3-methylanthranilate
MMA-4, methyl 4-methylanthranilate
MMA-5, methyl 5-methylanthranilate
MMA-6, methyl 6-methylanthranilate

A1. Computational Studies

Theoretical calculations of the molecular structures were performed using the Gaussian-09 software [1].

This section presents four tables, labeled from S1 to S4. These tables contain the absolute standard enthalpies, $H^\circ_{298.15\ \mathrm{K}}$, and entropies, $S^\circ_{298.15\ \mathrm{K}}$, obtained using the G3(MP2)//B3LYP [2] composite method for each of the methyl methylanthranilate isomers. Additionally, the corresponding derived gas-phase standard molar enthalpies, $\Delta_\mathrm{f}H^\circ_\mathrm{m}(\mathrm{g})$, entropies, $\Delta_\mathrm{f}S^\circ_\mathrm{m}(\mathrm{g})$, and Gibbs energy of formation, $\Delta_\mathrm{f}G^\circ_\mathrm{m}(\mathrm{g})$, are provided at T = 298.15 K, along with the conformational composition, χ_i.

In tables A1 to A4:

- The derived gas-phase standard molar enthalpies of formation, $\Delta_\mathrm{f}H^\circ_\mathrm{m}(\mathrm{g})$, were estimated from 7 reactions presented in Table 1 of manuscript.

- The absolute standard entropies, $S^\circ_{298.15\mathrm{K}}$, were obtained from B3LYP/6-31G(d) method for a frequency factor scale of 1.0029 [3];

- The gas-phase standard molar entropies of formation were calculated from $\Delta_\mathrm{f}S^\circ_\mathrm{m}(\mathrm{g}) = S^\circ_{298.15\mathrm{K}}\,(\text{conformer } i) - \sum S^\circ_{298.15\mathrm{K}}\,(\text{elements})$, considering the standard absolute entropy elements values, at 298.15 K, $S^\circ_{298.15\mathrm{K}}\left(\mathrm{H_2, g}\right)$ = 130.680 J·K^{-1}·mol^{-1}, $S^\circ_{298.15\mathrm{K}}\left(\mathrm{C, graphite}\right)$ = 5.740 J·K^{-1}·mol^{-1}, $S^\circ_{298.15\mathrm{K}}\left(\mathrm{N_2, g}\right)$ = 191.61 J·K^{-1}·mol^{-1}, and $S^\circ_{298.15\mathrm{K}}\left(\mathrm{O_2, g}\right)$ = 205.147 J·K^{-1}·mol^{-1} taken from ref. [4];

- The gas-phase standard molar Gibbs energy of formation were calculated from $\Delta_\mathrm{f}G^\circ_\mathrm{m}(\mathrm{g}) = \Delta_\mathrm{f}H^\circ_\mathrm{m}(\mathrm{g}) - T\Delta_\mathrm{f}S^\circ_\mathrm{m}(\mathrm{g})$;

- The conformational composition, χ_i, for each conformer was calculated assuming a Boltzmann distribution of the n possible equilibrium structures, $\chi_i = e^{-[\Delta_\mathrm{f}G^\circ_\mathrm{m}(\mathrm{g})/RT]}/\sum_i^n e^{-[\Delta_\mathrm{f}G^\circ_\mathrm{m}(\mathrm{g})/RT]}$.

Table S1. Absolute standard enthalpies, $H^{\circ}_{298.15\ K}$, and entropies, $S^{\circ}_{298.15\ K}$, obtained by G3(MP2)//B3LYP composite method for MMA-3 and the corresponding derived gas-phase standard molar enthalpies, $\Delta_f H^{\circ}_m$(g), entropies, $\Delta_f S^{\circ}_m$(g), and Gibbs energy of formation, $\Delta_f G^{\circ}_m$(g), at T = 298.15 K, and the conformational composition, χ_i.

Conformations of MMA-3 [1]		$H^{\circ}_{298.15K}$ [2] /a.u.	$\Delta_f H^{\circ}_m$(g) / kJ·mol⁻¹	$S^{\circ}_{298.15K}$ / J·K⁻¹·mol⁻¹	$\Delta_f S^{\circ}_m$(g) / J·K⁻¹·mol⁻¹	$\Delta_f G^{\circ}_m$(g) / kJ·mol⁻¹	χ_i
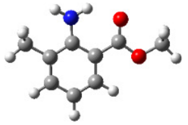 (1)	**(1)**	–553.960367	–321.6 ± 2.9	436.00	–635.4	–132.2	0.496
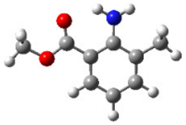 (2)	**(2)**	–553.960366	–321.6 ± 2.9	436.00	–635.4	–132.2	0.496
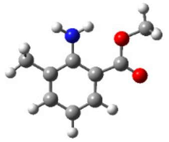 (3)	**(3)**	–553.955693	–309.3 ± 2.9	438.00	–633.4	–120.5	0.004
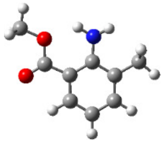 (4)	**(4)**	–553.955693	–309.3 ± 2.9	438.00	–633.4	–120.5	0.004

[1]Spheres color code: grey, C; red, O; blue, N; white, H.
[2] 1 a. u. (Hartree) corresponds to 2625.50 kJ·mol⁻¹.

Table S2. Absolute standard enthalpies, $H^{\circ}_{298.15\ K}$, and entropies, $S^{\circ}_{298.15\ K}$, obtained by G3(MP2)//B3LYP composite method for MMA-4 and the corresponding derived gas-phase standard molar enthalpies, $\Delta_f H^{\circ}_m$(g), entropies, $\Delta_f S^{\circ}_m$(g), and Gibbs energy of formation, $\Delta_f G^{\circ}_m$(g), at T = 298.15 K, and the conformational composition, χ_i.

Conformations of MMA-4 [1]		$H^{\circ}_{298.15K}$ [2] / a.u.	$\Delta_f H^{\circ}_m$(g) / kJ·mol⁻¹	$S^{\circ}_{298.15K}$ / J·K⁻¹·mol⁻¹	$\Delta_f S^{\circ}_m$(g) / J·K⁻¹·mol⁻¹	$\Delta_f G^{\circ}_m$(g) / kJ·mol⁻¹	χ_i
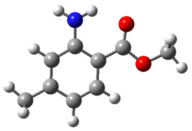 (1)	**(1)**	–553.960190	–322.7 ± 3.2	449.94	–621.4	–137.4	0.495
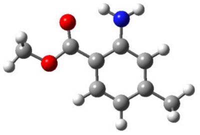 (2)	**(2)**	–553.960191	–322.7 ± 3.2	449.91	–621.4	–137.4	0.493
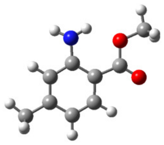 (3)	**(3)**	–553.955878	–311.4 ± 3.2	451.38	–620.0	–126.6	0.006
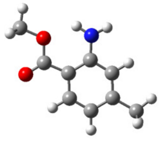 (4)	**(4)**	–553.955877	–311.4 ± 3.2	451.36	–620.0	–126.5	0.006

[1]Spheres color code: grey, C; red, O; blue, N; white, H.
[2] 1 a. u. (Hartree) corresponds to 2625.50 kJ·mol⁻¹.

Table S3. Absolute standard enthalpies, $H^{\circ}_{298.15\ K}$, and entropies, $S^{\circ}_{298.15\ K}$, obtained by G3(MP2)//B3LYP composite method for MMA-5 and the corresponding derived gas-phase standard molar enthalpies, $\Delta_f H^{\circ}_m(g)$, entropies, $\Delta_f S^{\circ}_m(g)$, and Gibbs energy of formation, $\Delta_f G^{\circ}_m(g)$, at T = 298.15 K, and the conformational composition, χ_i.

**Conformations of MMA-5** [1]			$H^{\circ}_{298.15K}$ [2] / a.u.	$\Delta_f H^{\circ}_m(g)$ / kJ·mol^{-1}	$S^{\circ}_{298.15K}$ / J·K^{-1}·mol^{-1}	$\Delta_f S^{\circ}_m(g)$ / J·K^{-1}·mol^{-1}	$\Delta_f G^{\circ}_m(g)$ / kJ·mol^{-1}	χ_i
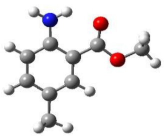 **(1)**	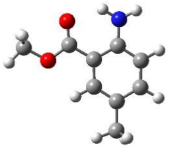 **(2)**	**(1)**	–553.958644	–314.1 ± 6.7	449.30	–622.1	–128.6	0.494
		**(2)**	–553.958643	–314.1 ± 6.7	449.30	–622.1	–128.6	0.494
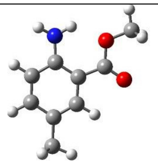 **(3)**	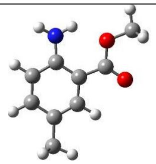 **(4)**	**(3)**	–553.954551	–303.3 ± 6.7	448.80	–622.6	–117.7	0.006
		**(4)**	–553.954551	–303.3 ± 6.7	448.80	–622.6	–117.7	0.006

[1]Spheres color code: grey, C; red, O; blue, N; white, H.
[2] 1 a. u. (Hartree) corresponds to 2625.50 kJ·mol^{-1}.

Table S4. Absolute standard enthalpies, $H^{\circ}_{298.15\ K}$, and entropies, $S^{\circ}_{298.15\ K}$, obtained by G3(MP2)//B3LYP composite method for MMA-6 and the corresponding derived gas-phase standard molar enthalpies, $\Delta_f H^{\circ}_m(g)$, entropies, $\Delta_f S^{\circ}_m(g)$, and Gibbs energy of formation, $\Delta_f G^{\circ}_m(g)$, at T = 298.15 K, and the conformational composition, χ_i.

**Conformations of MMA-6** [1]			$H^{\circ}_{298.15K}$ [2] / a.u.	$\Delta_f H^{\circ}_m(g)$ / kJ·mol^{-1}	$S^{\circ}_{298.15K}$ / J·K^{-1}·mol^{-1}	$\Delta_f S^{\circ}_m(g)$ / J·K^{-1}·mol^{-1}	$\Delta_f G^{\circ}_m(g)$ / kJ·mol^{-1}	χ_i [7]
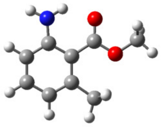 **(1)**	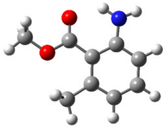 **(2)**	**(1)**	–553.953032	–303.9 ± 3.2	439.42	–631.9	–115.5	0.457
		**(2)**	–553.953032	–303.9 ± 3.2	439.41	–631.9	–115.5	0.457
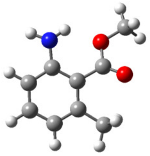 **(3)**	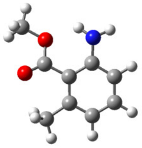 **(4)**	**(3)**	–553.950919	–298.4 ± 3.2	437.96	–633.4	–109.6	0.042
		**(4)**	–553.950910	–298.4 ± 3.2	438.38	–633.0	–109.7	0.044

[1]Spheres color code: grey, C; red, O; blue, N; white, H.
[2] 1 a. u. (Hartree) corresponds to 2625.50 kJ·mol^{-1}.

Table S5. G3(MP2)//B3LYP enthalpies with corresponding gas-phase standard (p^o = 0.1 MPa) molar enthalpies of formation, at T = 298.15 K, for the atomic and molecular species.

**Atom**	**Formula**	$H^{\circ}_{298.15K}$ **/ a. u. (χi)[1,2]**	$\Delta_f H^o_m$**(g) /kJ·mol-1**
Carbon	C	–37.788425	716.67 [4]
Hydrogen	H	–0.499780	218.00 [4]
Oxygen	O	–74.989704	249.17 [4]
Nitrogen	N	–54.524582	472.68 [4]
**Compound**	**Formula**	$H^{\circ}_{298.15K}$ **/ a. u. (χi)[a]**	$\Delta_f H^o_m$**(g) /kJ·mol-1**
Benzene	C_6H_6	–231.835164	82.6 ± 0.7 [5]
Butane	C_4H_{10}	–158.120780	–125.7 ± 0.6 [5]
Butyl acetate	$C_6H_{12}O_2$	–385.715515 (**0.39**)<br>–385.715140 (**0.17**)<br>–385.715097 (**0.11**)<br>–385.715001 (**0.09**)<br>–385.714103 (**0.05**)<br>–385.714777 (**0.05**)<br>–385.714395 (**0.05**)<br>–385.713873 (**0.02**)<br>–385.713831 (**0.02**)<br>–385.713265 (**0.02**)<br>–385.713263 (**0.02**)	–485.3 ± 0.7 [5]
Ethane	C_2H_6	–79.651016	–83.8 ± 0.3 [5]
Ethyl acetate	$C_4H_8O_2$	–307.245657 (**0.71**)<br>–307.245239 (**0.29**)	–443.6 ± 0.5 [5]
Methane	CH_6	–40.420547	–74.4 ± 0.4 [5]
Methyl acetate	$C_3H_6O_2$	–268.006106	–413.3 ± 0.7 [5]
Methyl anthranilate	$C_8H_9NO_2$	–514.720048 (**0.484**)<br>–514.720042 (**0.502**)<br>–514.715856 (**0.007**)<br>–514.715850 (**0.007**)	–284.1 ± 3.0 [6]
Methyl benzoate	$C_8H_8O_2$	–459.430197	–273.45 ± 0.77 [7]
Methyl 3-methylanthranilate	$C_9H_{11}NO_2$	–553.960367 (**0.496**)<br>–553.960366 (**0.496**)<br>–553.955693 (**0.004**)<br>–553.955693 (**0.004**)	
Methyl 4-methylanthranilate	$C_9H_{11}NO_2$	–553.960190 (**0.495**)<br>–553.960191 (**0.493**)<br>–553.955878 (**0.006**)<br>–553.955877 (**0.006**)	

…/…

Table S5. (continuation)

Compound	Chemical Structure	$H^{\circ}_{298.15K}$ / a. u. (χ_i)[1,2]	$\Delta_f H^o_m(g)$ /kJ·mol^{-1}
Methyl 5-methylanthranilate	$C_9H_{11}NO_2$	−553.958644 (**0.494**)<br>−553.958643 (**0.494**)<br>−553.954551 (**0.006**)<br>−553.954555 (**0.006**)	
Methyl 6-methylanthranilate	$C_9H_{11}NO_2$	−553.953037 (**0.457**)<br>−553.953037 (**0.457**)<br>−553.950919 (**0.042**)<br>−553.950910 (**0.044**)	–
Propane	C_3H_8	−118.885800	−104.7 ± 0.5 [5]
Propyl acetate	$C_5H_{10}O_2$	−346.480526 (**0.39**)<br>−346.480253 (**0.31**)<br>−346.480105 (**0.16**)<br>−346.480093 (**0.14**)	−464.8 [8]
Toluene	C_7H_8	−271.074388	50.5 ± 0.5 [5]
o-Toluidine	$C_7H_9N_2$	−326.360243	53.2 ± 0.5 [9]
m-Toluidine	$C_7H_9N_2$	−326.359301	53.4
p-Toluidine	$C_7H_9N_2$	−326.358419	62.2 [10]

[1] χ_i corresponds to the conformer composition.
[2] 1 a. u. (Hartree) corresponds to 2625.50 kJ·mol^{-1}.

S2. Combustion Calorimetry

Tables S6 and S7 display the detailed combustion results for the MMA-3 and MMA-5 isomers.

The symbols presented in tables have the following meaning:
- $m(CO_2$, total), mass of carbon dioxide;
- *m*(cpd), mass of compound corrected for air buoyancy;
- *m*(cpd corrected), mass of compound considering the % CO_2 recovery;
- *m*(fuse), mass of fuse (cotton);
- *m*(Melinex®), mass of Melinex, corrected for water content;
- T_i, initial temperature increase;
- T_f, final temperature increase;
- ΔT_{ad}, corrected temperature rise;
- ε_f, energy equivalent for the contents in the final state;
- $\Delta m(H_2O)$, deviation in mass of water added to the calorimeter from 3116.6 g;
- ΔU(IBP), internal energy associated with the isothermal combustion reaction under actual bomb conditions;
- ΔU(Melinex®), energy of combustion of Melinex;
- ΔU(fuse), energy of combustion of the fuse (cotton);
- $\Delta U(HNO_3)$, energy correction for nitric acid formation;
- ΔU(ign), electric energy for ignition;
- ΔU_Σ, standard state correction;
- $\Delta_c u^o$ (corrected), standard ($p° = 0.1$ MPa) massic energy of combustion for the compound, determined from the compound mass based on carbon dioxide recovery;
- % CO_2, percentage of recovered carbon dioxide.

Table S6 Combustion results and standard ($p°$ = 0.1 MPa) massic energy of combustion, at T = 298.15 K, for MMA-3.

Experiment	1	2	3	4	5	6
m (CO_2, total) / g	1.53843	1.51258	1.51736	1.55050	1.44468	1.53261
m (cpd) / g	0.57664	0.56755	0.56786	0.58147	0.57003	0.57026
m (cpd corrected) / g	0.57711	0.56797	0.56823	0.58131	0.57016	0.57015
m (fuse) / g	0.00226	0.00227	0.00211	0.00230	0.00217	0.00224
m (Melinex®) / g	0.6593	0.06421	0.06613	0.06677	0.03234	0.07069
m (carbon) / g	0	0	0	0	0	0
T_i / K	298.15007	298.15124	298.15115	298.15046	298.15084	298.14981
T_f / K	299.35195	299.33516	299.33797	299.36168	299.29447	299.34700
ΔT_{ad} / K	1.12689	1.10813	1.11156	1.13668	1.06616	1.12158
ε_f / (J·K^{-1})	17.10	17.06	17.07	17.11	17.01	17.09
Δm (H_2O) / g	0	-0.1	0	0	0	0
$-\Delta U$ (IBP) / J	18051.76	17750.21	17805.93	18208.69	17078.51	17966.17
ΔU (carbon) / J	0	0	0	0	0	0
$-\Delta U$ (Melinex®) / J	1510.04	1470.52	1514.61	1529.23	740.63	1619.01
$-\Delta U$ (fuse) / J	36.70	36.86	34.27	37.35	35.24	36.38
$-\Delta U$ (HNO_3) / J	27.25	29.75	30.10	29.11	27.13	28.88
ΔU (ign) / J	0.68	1.19	0.89	0.59	0.96	1.19
ΔU_Σ / J	10.71	10.49	10.53	10.81	9.90	10.68
$-\Delta_c u°$ (corrected)/ (J·g^{-1})	28533.66	28527.19	28538.48	28559.96	28528.15	28538.49
% CO_2	100.081	100.074	100.066	99.973	100.023	99.981
$\langle -\Delta_c u° \rangle$ = (28537.7 ±4.9)[1] J·g^{-1}						

[1] The uncertainty corresponds to the estimated standard deviation of the mean for six experiments.

TABLE S7 Combustion results and standard ($p° = 0.1$ MPa) massic energy of combustion, at $T = 298.15$ K, for MMA-5.

Experiment	1	2	3	4	5	6
m (CO_2, total) / g	1.53965	1.27253	1.71297	1.63147	1.38646	1.86482
m (cpd) / g	0.64057	0.52934	0.71288	0.67925	0.57664	0.77687
m (cpd corrected) / g	0.64049	0.52922	0.71302	0.67893	0.57673	0.77628
m (fuse) / g	0.00241	0.00222	0.00204	0.00220	0.00223	0.00215
T_i / K	298.15056	298.15035	298.15036	298.15356	298.14961	298.15069
T_f / K	299.36901	299.17828	299.49163	299.43772	299.25826	299.60399
ΔT_{ad} / K	1.14371	0.94545	1.27117	1.21239	1.02947	1.38397
ε_f / ($J \cdot K^{-1}$)	17.42	17.12	17.63	17.53	17.24	17.80
Δm (H_2O) / g	0.2	0	0	0	0	0
$-\Delta U$ (IBP) / J	18322.09	15144.66	20363.24	19421.49	16490.74	22170.55
$-\Delta U$ (fuse) / J	39.14	36.05	33.13	35.73	36.22	34.92
$-\Delta U$ (HNO_3) / J	31.69	25.29	32.51	32.45	30.69	36.09
ΔU (ign) / J	1.12	1.18	1.20	1.16	1.20	1.20
ΔU_Σ / J	10.49	8.52	11.82	11.22	9.33	13.01
$-\Delta_c u^o$ (corrected)/ ($J \cdot g^{-1}$)	28479.40	28484.94	28450.51	28489.08	28461.33	28451.76
% CO_2	99.988	99.977	100.020	99.953	100.014	99.921
$\langle -\Delta_c u^o \rangle = (28469.5 \pm 7.0)$[1] $J \cdot g^{-1}$ [c]						

[1]The uncertainty corresponds to the estimated standard deviation of the mean for six experiments.

A3. Vacuum drop microcalorimetric technique

Tables S8 and S9 show the detailed results obtained from Calvet microcalorimetry for the vaporization process of MMA-3 and the sublimation process of MMA-5.

The symbols presented in tables have the following meaning:

- T_{Calvet}, temperature of the hot reaction vessel;
- m(sct) mass of the sample capillary tube;
- m(rct) mass of the reference capillary tube;
- m(cpd), mass of the compound;
- ΔH(blank), blank heat capacity corrections for the glass capillary tubes;
- ΔH(total), total enthalpy calculated from the area of the enthalpic peak obtained in the experiment;
- ΔH(corr), enthalpy change corrected taking into account the blank experiments;
- $\Delta_{\mathrm{l},\,298.15\mathrm{K}}^{\mathrm{g},\,T_{\mathrm{Calvet}}} H_{\mathrm{m}}^{\circ}$ (exp), enthalpy of vaporization from 298.15 K to temperature of the hot reaction vessel;
- $\Delta_{\mathrm{cr},\,298.15\mathrm{K}}^{\mathrm{g},\,T_{\mathrm{Calvet}}} H_{\mathrm{m}}^{\circ}$ (exp), enthalpy of sublimation from 298.15 K to temperature of the hot reaction vessel;
- $\Delta_{298.15\,\mathrm{K}}^{T_{\mathrm{Calvet}}} H_{\mathrm{m}}^{\circ}$ (g), enthalpy change in the gas-phase phase from 298.15 K to the temperature of the hot reaction vessel;
- $\Delta_{\mathrm{l}}^{\mathrm{g}} H_{\mathrm{m}}^{\circ}$ (298.15 K), enthalpy of vaporization at 298.15 K of the compound;
- $\Delta_{\mathrm{cr}}^{\mathrm{g}} H_{\mathrm{m}}^{\circ}$ (298.15 K), enthalpy of sublimation at 298.15 K of the compound.

TABLE S8 Calvet microcalorimetry results for the process of vaporization of MMA-3.

Experiment	1	2	3	4	5	6
T_{Calvet} / K	345.85	345.58	345.70	345.46	345.58	345.72
m(sct) / mg	19.4729	19.5920	20.2518	19.4882	20.5738	20.0952
m(rct) / mg	19.5142	19.5986	20.3113	19.5908	20.6461	20.1245
m(cpd) / mg	6.449	5.263	5.286	5.852	6.653	5.680
ΔH(blank) / mJ	32.531	31.522	32.869	34.758	33.057	31.969
ΔH(total) / J	3.034	2.463	2.489	2.763	3.117	2.669
ΔH(corr) / J	3.067	2.495	2.522	2.798	3.150	2.701
$\Delta_{\mathrm{l},\,298.15\mathrm{K}}^{\mathrm{g},\,T_{\mathrm{Calvet}}} H_{\mathrm{m}}^{\circ}$ (exp) / kJ·mol^{-1}	80.83	80.57	81.11	81.26	80.48	80.81
$\Delta_{298.15\,\mathrm{K}}^{T_{\mathrm{Calvet}}} H_{\mathrm{m}}^{\circ}$ (g) / kJ·mol^{-1}	10.037	9.977	10.004	9.950	9.977	10.008
$\Delta_{\mathrm{l}}^{\mathrm{g}} H_{\mathrm{m}}^{\circ}$ (298.15 K) / kJ·mol^{-1}	70.79	70.59	71.10	71.31	70.50	70.80
$\Delta_{\mathrm{l}}^{\mathrm{g}} H_{\mathrm{m}}^{\circ}$ (298.15 K) = 70.8 ± 2.0[1] kJ·mol^{-1}						

[1]The quoted uncertainty corresponds to the expanded uncertainty determined from the combined standard uncertainty (which include the contribution of calibration with decane) and the coverage factor k = 2.16 (for an effective degrees of freedom of 13, calculated from Welch-Satterthwaite formula, and a 0.95 level of confidence) [11].

TABLE S9 Calvet microcalorimetry results for the process of sublimation of MMA-5.

Experiment	1	2	3	4	5	6
T_{Calvet} / K	333.52	333.52	333.65	333.52	333.56	333.65
m(sct) / mg	21.0344	22.0145	22.1685	21.8166	21.5491	20.2036
m(rct) / mg	21.0983	22.0612	22.1809	21.9153	21.5831	20.3106
m(cpd) / mg	4.360	3.610	5.565	3.356	3.766	3.627
ΔH(blank) / mJ	30.140	29.227	28.318	30.616	29.149	31.652
ΔH(total) / J	2.335	2.024	3.120	1.826	2.059	2.007
ΔH(corr) / J	2.365	2.053	3.148	1.856	2.088	2.039
$\Delta_{\mathrm{cr,\ 298.15K}}^{\mathrm{g,}\ T_{\mathrm{Calvet}}} H_{\mathrm{m}}^{\circ}$ (exp) / kJ·mol^{-1}	94.66	99.26	98.72	96.53	96.76	98.10
$\Delta_{298.15\ \mathrm{K}}^{T_{\mathrm{Calvet}}} H_{\mathrm{m}}^{\circ}$ (g) / kJ·mol^{-1}	7.38	7.38	7.41	7.38	7.39	7.41
$\Delta_{\mathrm{cr}}^{\mathrm{g}} H_{\mathrm{m}}^{\circ}$ (298.15 K) / kJ·mol^{-1}	87.28	91.89	91.31	89.15	89.38	90.69
$\Delta_{\mathrm{cr}}^{\mathrm{g}} H_{\mathrm{m}}^{\circ}$ (298.15 K) = 89.9 ± 2.0[1] kJ·mol^{-1} [c]						

[1]The standard uncertainty corresponds to the expanded uncertainty determined from the combined standard uncertainty (which include the contribution of calibration with naphthalene) and the coverage factor k = 2.23 (for an effective degrees of freedom of 11, calculated from Welch-Satterthwaite formula, and a 0.95 level of confidence) [11].

Table S10 Standard (p° = 0.1 MPa) molar heat capacities in the gaseous phase for MMA-3 and MMA-5, obtained from statistical thermodynamics using the vibrational frequencies calculated at the B3LYP/6-31G(d) level of theory (scaled by a factor of 0.960 ± 0.022) [12].

T / K	C_p^{o}(g) / J·K^{-1}·mol^{-1}	
	MMA-3	MMA-5
200.00	143.42	146.00
250.00	171.18	173.17
298.15	197.62	199.18
300.00	198.63	200.17
350.00	225.19	226.40
400.00	250.25	251.21
450.00	273.40	274.17
500.00	294.50	295.13
550.00	313.61	314.13
600.00	330.90	331.33

Polynomial functions were obtained for MMA-3 and MMA-5 from the use of the tabulated values. Mathematical expressions resulted from these and are present in eqs. S1 and S2.

C_p^o (MMA-3, g) / (J·K^{-1}·mol^{-1}) = –3.65641 × 10^{-7} (T / K)^3 + 1.13865 × 10^{-4} (T / K)^2 +
+ 5.67683 × 10^{-1} (T / K) + 28.0733 (r^2 = 0.9999) (S1)

C_p^o (MMA-5, g) / (J·K^{-1}·mol^{-1}) = –3.92751× 10^{-7} (T / K)^3 + 1.60091× 10^{-4} (T / K)^2 +
+ 5.39425 × 10^{-1} (T / K) + 34.6683 (r^2 = 0.9999) (S2)

S4. Knudsen Mass-Loss Effusion Method

The results for the sublimation experiments obtained for MMA-5 are given in the Table S11. The symbols presented have the following meaning:

-*T* is the selected temperature of the aluminium block;
- *t* is the effusion period;
- *m* is the mass effused and *ρ* is the vapor pressure.
- the subscripts S, M and L correspond to the small, medium and large effusion orifices respectively.

The areas, A_0, for each orifice are: A_0(S1,S2,S3) = (0.636 ± 0.004) mm^2, A_0(M4,M5,M6) = (0.785 ± 0.004) mm^2, and A_0(L7,L8,L9) = (0.985 ± 0.004) mm^2;

The Clausing factors, ω_0, for each orifice are: ω_0(S1,S2,S3) = 0.986, ω_0 (M4,M5,M6) = 0.988, and ω_0 (L7,L8,L9) = 0.989.

TABLE S11 Knudsen mass-loss effusion results for MMA-5 at different orifices areas.

T/K[1]	*t*/s	Cells	*m* /mg			*p* /Pa[2]		
			m_S	m_M	m_L	p_S	p_M	p_L
293.40	30063	S3-M6-L9	9.64	11.68	14.97	0.1557	0.1526	0.1557
294.28	30063	S2-M5-L8	11.01	13.24	16.75	0.1781	0.1732	0.1745
295.80	22550	S2-M5-L8	9.85	11.91	15.05	0.2129	0.2083	0.2095
297.79	18839	S2-M5-L8	10.61	12.85	16.22	0.2754	0.2698	0.2712
298.80	28879	S2-M5-L8	18.68	22.47	28.19	0.3169	0.3083	0.3080
300.12	28879	S1-M4-L7	22.35	27.15	33.60	0.3800	0.3734	0.3679
301.61	11915	S1-M4-L7	10.84	13.14	16.47	0.4478	0.4391	0.4382
303.29	11076	S2-M5-L8	12.31	14.67	18.47	0.5486	0.5288	0.5301
304.61	11076	S1-M4-L7	14.66	17.63	22.00	0.6547	0.6369	0.6327
306.26	11915	S2-M5-L8	18.71	22.40	28.22	0.7789	0.7543	0.7565
307.76	10779	S2-M5-L8	20.39	24.63	30.73	0.9405	0.9190	0.9129
309.09	18839	S1-M4-L7	41.55	50.67	63.28	1.0990	1.0841	1.0779

[1]The standard uncertainty of the measured temperature is ± (1·10^{-2}) K;
[2]The standard uncertainty is ± (3·10^{-2}) Pa.

The standard ($p°$ = 0.1 MPa) molar enthalpy of sublimation, at the mean temperature of the experimental range, $\Delta_{\mathrm{cr}}^{\mathrm{g}}H_{\mathrm{m}}^{\circ}(\langle T\rangle)$, was calculated through the integrated form of the Clausius–Clapeyron equation, ln(p/Pa) = $a - b$·(K/T), where a is a constant and $b = \Delta_{\mathrm{cr}}^{\mathrm{g}}H_{\mathrm{m}}^{\mathrm{o}}(\langle T\rangle)/R$ (R = 8.3144598 J·K^{-1}·mol^{-1}), and the standard molar entropy of sublimation at the equilibrium pressure and mean temperature, $\Delta_{\mathrm{cr}}^{\mathrm{g}}S_{\mathrm{m}}\big(\langle T\rangle, p(\langle T\rangle)\big)$, calculated by eq. A3.

$$\Delta_{\mathrm{cr}}^{\mathrm{g}}S_{\mathrm{m}}\big(\langle T\rangle, p(\langle T\rangle)\big)\ /\ \mathrm{J{\cdot}K^{-1}{\cdot}mol^{-1}} = \Delta_{\mathrm{cr}}^{\mathrm{g}}H_{\mathrm{m}}^{\circ}(\langle T\rangle)/\langle T\rangle \qquad \text{(S3)}$$

The values of the standard molar enthalpy, entropy and Gibbs energy of sublimation, at T = 298.15 K, were calculated using eqs. S4 to S6, respectively. The value of the difference in standard molar heat capacity at constant pressure between the gaseous and the crystalline phase used for MMA-5 was, $\Delta_{\mathrm{cr}}^{\mathrm{g}}C_{p,\mathrm{m}}^{\circ}$ = –36.0 J·K^{-1}·mol^{-1}.. Theis value was calculated through eq. S7 derived by Monte et al. [13] as a rearrangement of eq. A8 suggested by Chickos and collaborators [14], using the respective value of the standard molar heat capacity at constant pressure in the gaseous phase at T = 298.15 K, derived from statistical thermodynamics using the vibrational frequencies calculated at the B3LYP/6-31G(d) level of theory, scaled by a factor of 0.960 ± 0.022 [12], reported in Table S10.

$$\Delta_{\mathrm{cr}}^{\mathrm{g}}H_{\mathrm{m}}^{\circ}(298.15\mathrm{K})/\mathrm{kJ{\cdot}mol^{-1}} = \Delta_{\mathrm{cr}}^{\mathrm{g}}H_{\mathrm{m}}^{\circ}(\langle T\rangle) - \Delta_{\mathrm{cr}}^{\mathrm{g}}C_{p,\mathrm{m}}^{\circ}(\langle T\rangle - 298.15) \qquad \text{(S4)}$$

$$\Delta_{\mathrm{cr}}^{\mathrm{g}}S_{\mathrm{m}}^{\circ}(298.15\mathrm{K})/\mathrm{J{\cdot}K^{-1}{\cdot}mol^{-1}} = \Delta_{\mathrm{cr}}^{\mathrm{g}}S_{\mathrm{m}}^{\circ}(\langle T\rangle, p\langle T\rangle) + R\ln[p(\langle T\rangle)/p°] + \Delta_{\mathrm{cr}}^{\mathrm{g}}C_{p,\mathrm{m}}^{\circ}\ln(298.15/\langle T\rangle) \qquad \text{(S5)}$$

$$\Delta_{\mathrm{cr}}^{\mathrm{g}}G_{\mathrm{m}}^{\circ}(298.15\mathrm{K})/\ \mathrm{kJ{\cdot}mol^{-1}} = \Delta_{\mathrm{cr}}^{\mathrm{g}}H_{\mathrm{m}}^{\circ}(298.15) - 298.15\cdot\Delta_{\mathrm{cr}}^{\mathrm{g}}S_{\mathrm{m}}^{\circ}(298.15) \qquad \text{(S6)}$$

$$\Delta_{cr}^{g}C_{p,\mathrm{m}}^{\circ}/\mathrm{J\cdot K^{-1}\cdot mol^{-1}} = -\{0.9 + 0.176\ C_{p,\mathrm{m}}^{\circ}(g)\} \qquad \text{(S7)}$$

$$\Delta_{cr}^{g}C_{p,\mathrm{m}}^{\circ}/\mathrm{J\cdot K^{-1}\cdot mol^{-1}} = -\{0.75 + 0.15\ C_{p,\mathrm{m}}^{\circ}(cr)\} \qquad \text{(S8)}$$

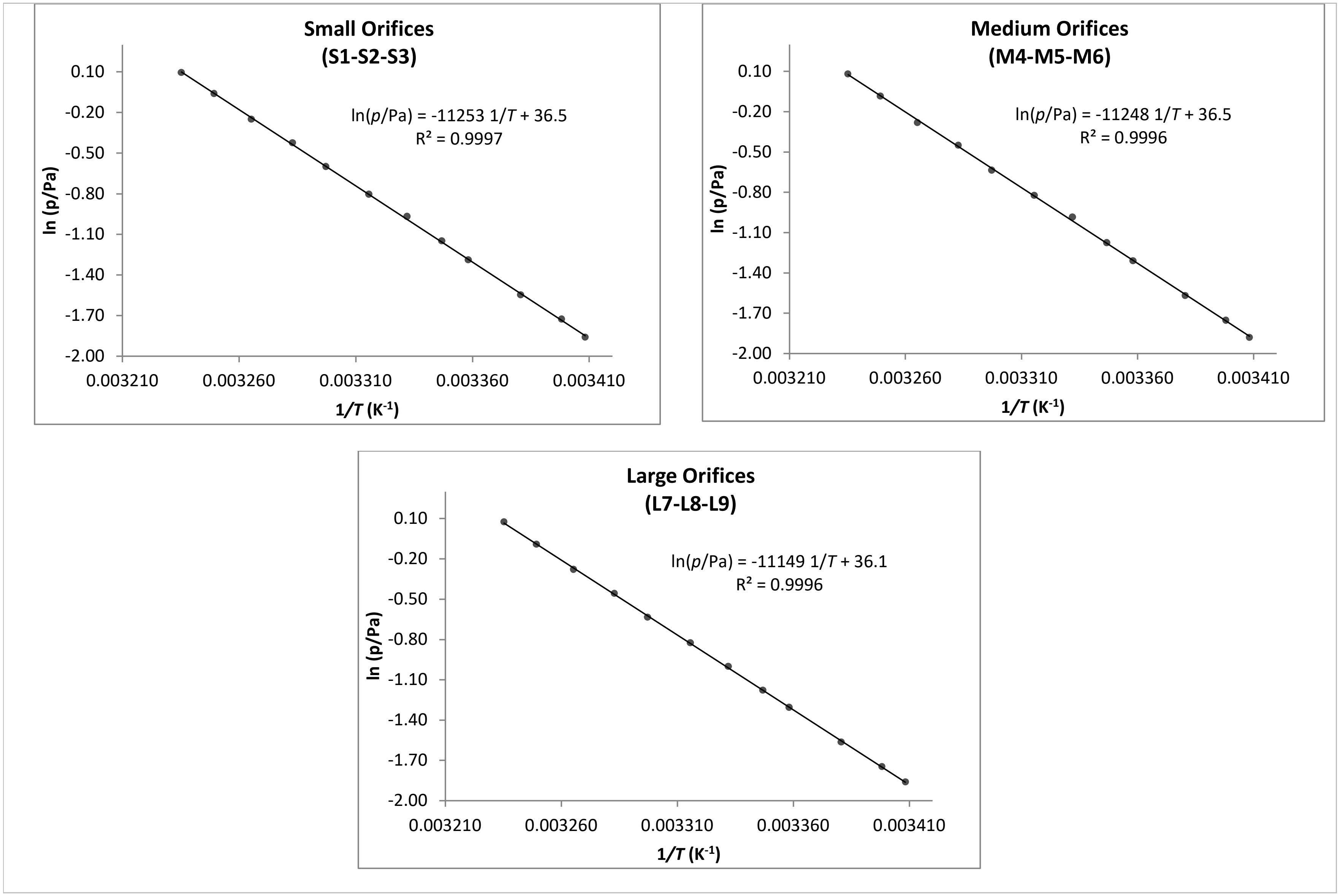

Figure S1. Plots of ln(p/Pa) against 1/T for small, medium and large orifices obtained by the mass-loss Knudsen effusion experiments for MMA-5.

S5. Differential Scanning Calorimetry

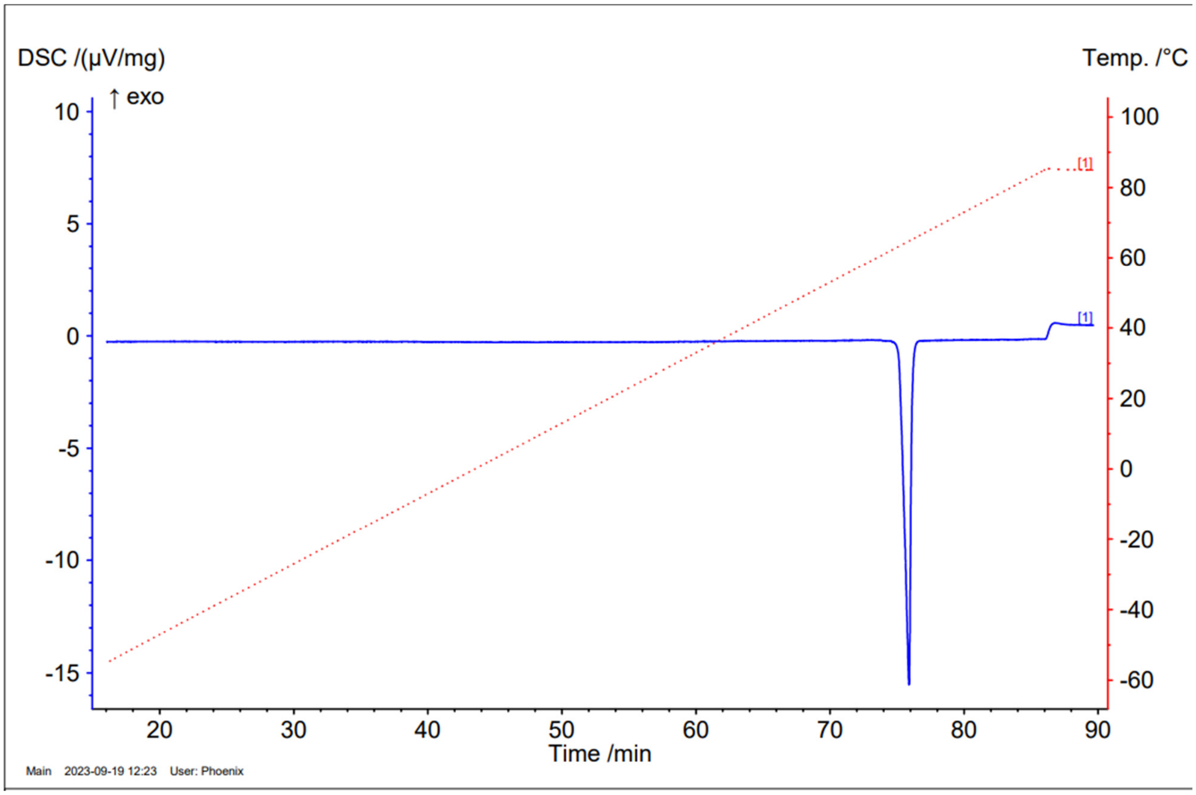

Figure S2. DSC curve obtained for MMA-5

References

1. Gaussian 09, Revision A.02, Frisch, M.J.; Trucks, G.W.; Schlegel H.B.; Scuseria, G.E.; Robb, M.A.; Cheeseman, J.R.; Scalmani, G.; Barone, V.; Petersson, G.A.; Nakatsuji, H.; Li, X.; Caricato, M.; Marenich, A.; Bloino, J.; Janesko, B.G.; Gomperts, R.; Mennucci, B.; Hratchian, H.P.; Ortiz, J.V.; Izmaylov, A.F.; Sonnenberg, J.L.; Williams-Young, D.; Ding, F.; Lipparini, F.; Egidi, F.; Goings, J.; Peng, B.; Petrone, A.; Henderson, T.; Ranasinghe, D.; Zakrzewski, V.G.; Gao, J.; Rega, N.; Zheng, G.; Liang, W.; Hada, M.; Ehara, M.; Toyota, K.; Fukuda, R.; Hasegawa, J.; Ishida, M.; Nakajima, T.; Honda, Y.; Kitao, O.; Nakai, H.; Vreven, T.; Throssell, K.; Montgomery Jr., J.A.; Peralta, J.E.; Ogliaro, F.; Bearpark, M.; Heyd, J.J.; Brothers, E.; Kudin, K.N.; Staroverov, V.N.; Keith, T.; Kobayashi, R.; Normand, J.; Raghavachari, K.; Rendell, A.; Burant, J.C.; Iyengar, S.S.; Tomasi, J.; Cossi, M.; Millam, J.M.; Klene, M.; Adamo, C.; Cammi, R.; Ochterski, J.W.; Martin, R.L.; Morokuma, K.; Farkas, O.; Foresman, J.B.; Fox, D.J.; Gaussian, Inc., Wallingford CT, 2016.
2. Baboul, A.G.; Curtiss, L.A.; Redfern, P.C.; Raghavachari, K.; Gaussian-3 theory using density functional geometries and zero-point energies. *J. Chem. Phys.* **1999**, *110*, 7650-7657.
3. Merrick, P.; Moran, D.; Radom, L. An evaluation of harmonic vibrational frequency scale factor. *J. Phys. Chem.* A **2007**, *111*, 11683-11700.
4. Chase Jr. M.W., NIST-Janaf Thermochemical Tables. *J Phys Chem Ref Data* (1998) Monograph 9 (part I and II), 1-1951. Available online: https://janaf.nist.gov/ Accessed on 01 may 2021).
5. Pedley, J.B. *Thermochemical Data and Structures of Organic Compounds Thermodynamics*, Research Center, College Station, Texas, 1994.
6. Freitas, V.L.S.; Silva, C.A.O.; Ribeiro da Silva, M.D.M.C.; Thermochemical study of anthranilate derivatives: Effect of the size of the alkyl substituent. *J. Chem. Thermodyn.* **2021**, *158*, 106441.
7. Steele, W.V.; Chirico, R.D.; Cowell, A.B.; Knipmeyer, S.E.; Nguyen, A. Thermodynamic properties and ideal-gas enthalpies of formation for methyl benzoate, ethyl benzoate, (*R*)-(+)-limonene, *tert*-amyl methyl ether, *trans*-crotonaldehyde, and diethylene glycol. *J. Chem. Eng. Data* **2002**, *47*, 667-688.
8. Albahri, T.A.; Aljasmi, A.F. SGC method for predicting the standard enthalpy of formation of pure compounds from their molecular structures. *Thermochim. Acta* **2013**, *415*, 46-60.

9. Steele, V.W.; Chirico, R.D.; Nguyen, A.; Knipmeyer, S.E. The thermodynamic properties of 2-methylaniline and trans-(*r*,*S*)-decahydroquinoline. *J. Chem. Thermodyn*, **1994**, *26*, 515-544.
10. Draeger, J.A. Chemical thermodynamic properties of molecules that undergo inversion. II The methylanilines, *J. Chem. Thermodyn*. **1984**, *16*, 1067-1073..
11. Taylor, B.N.; Kuyatt, C.E. *Guidelines for evaluating and expressing the uncertainty of NIST measurement results*. NIST Technical Note 1297, 1994 Edition.
12. NIST Computational Chemistry Comparison and Benchmark Database, NIST Standard Reference Database Number 101, Release 16a, August 2013, Editor: Russell D. Johnson III. https://cccbdb.nist.gov/vibscalex.asp. Accessed 5 july 2023.
13. Monte, M.J.S.; Almeida, A.R.R.P.; Matos, M.A.R. Thermodynamic study on the sublimation of five aminomethoxybenzoic acids. *J. Chem. Eng. Data* **2010**, *55*, 419-423.
14. Chickos, J.S.; Hosseini, S.; Hesse, D.G.; Liebman, J.F. Heat capacity corrections to a standard state: a comparison of new and some literature methods for organic liquids and solids, *Struct. Chem*. **1993**, *4*, 271-278.
